# Legged Robot with Tensegrity Feature Bionic Knee Joint

**DOI:** 10.1002/advs.202411351

**Published:** 2025-02-03

**Authors:** Qi Wen, Meiling Zhang, Jianwei Sun, Weijia Li, Jinkui Chu, Zhenyu Wang, Songyu Zhang, Luquan Ren

**Affiliations:** ^1^ School of Mechatronic Engineering Changchun University of Technology Changchun 130012 China; ^2^ School of Mechanical Engineering Dalian University of Technology Dalian 116024 China; ^3^ Key Laboratory of Bionic Engineering Jilin University Changchun 130022 China

**Keywords:** adaptive structure, bionic knee joint, legged robot, tensegrity structure

## Abstract

Legged robots, designed to emulate human functions, have greatly influenced numerous sectors. However, the focus on continuously improving the joint motors and control systems of existing legged robots not only increases costs and complicates maintenance but also results in failure to accurately mimic the functionality of the human skeletal‒muscular system. This study introduces a bionic legged robot structure that leverages the tensegrity principle, drawing inspiration from the human leg's structural morphology and kinematic mechanisms. By designing a system that distinguishes between rolling and sliding movements, the human knee's variable instantaneous center of rotation (ICR), is successfully replicated showcasing its capabilities in achieving gait resemblance and vibration absorption. The tensegrity unit's features, including remarkable deformability, self‐recovery, and the four‐bar mechanism's singular position characteristic, alongside a rope unlocking mechanism reminiscent of human muscles, facilitate in situ compliance–rigid–compliance transitions of the knee joint without the need for knee joint motors, relying solely on ground contact through the foot. This innovation overcomes the conventional dependency of legged robots on joint motors, as the system requires only a single DC motor positioned at the hip joint and a straightforward control program to seamlessly execute a complete cycle of a single leg's movement.

## Graphical abstract

This study presents a tensegrity‐based bionic legged robot that is inspired by the biomechanical structure and movement mechanisms of the human leg. A rigid‐to‐flexible transition during walking is achieved via a four‐bar mechanism and rope‐driven knee‒foot linkage. A single motor at the hip joint and a basic control program perform the gait cycle for a single leg.

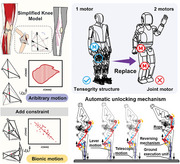

## 1 Introduction

The advent of robotics has revolutionized numerous industries, and robotics has assumed an increasingly pivotal role in our daily lives.^[^ ^1^, ^2^, ^3^ ^]^ Presently, two predominant types of robots are wheeled and legged varieties.^[^ ^4^ ^]^ Wheeled robots, characterized by their simplicity and efficiency on flat surfaces, have found widespread application in logistics, manufacturing, and exploration. Nevertheless, their mobility is constrained when navigating uneven or challenging terrains. In contrast, legged robots, which can emulate human or animal locomotion, exhibit enhanced maneuverability, and operational adaptability in complex environments.^[^ ^5^, ^6^, ^7^ ^]^ Research on legged robotics is diverse and innovative. For example, the bipedal robot ATRIAS uses a spring‐mass model to achieve dynamic walking.^[^ ^8^, ^9^ ^]^ The emergency response robot WANDERER achieves greater endurance through the use of motors and reduction gears.^[^ ^10^ ^]^ BRHL, a bipedal robot with artificial and intelligent bionic legs, is designed for an asymmetric gait.^[^ ^11^ ^]^ However, the aforementioned robotic systems employing multimotor configurations face significant challenges because of the resulting sharp increase in the complexity of their control systems. For example, multimotor setups often involve the placement of motors at the knee joint or the installation of hydraulic cylinders in the lower leg to achieve precise positioning and flexion. However, these systems lack inherent self‐recovery capabilities. Instead, they require additional signaling from the controller to restore the knee joint to its initial angle before transitioning to the next gait cycle. This undoubtedly increases system redundancy and computational demands.^[^ ^12^, ^13^ ^]^ Moreover, the majority of legged robots feature a single rotational joint at the knee, leading to a rigid gait that struggles to absorb impact loads, which affects the smoothness of the walking process.^[^ ^14^, ^15^ ^]^ Therefore, it is essential to develop more adaptable joint structures via alternative and improved technologies and to employ simplified control systems to increase the fluidity of legged robots during locomotion.^[^ ^16^ ^]^

Bionic legged robots, inspired by biological insights and principles of locomotion, are designed to emulate the characteristics and movement mechanisms of human legs. These designs leverage concepts from the study of natural locomotion systems. A human knee, comprising the femur, tibia, and ligaments, plays a crucial role in kinematic movements.^[^ ^17^ ^]^ Changes in the ICR due to patellar displacement significantly influence this movement.^[^ ^18^, ^19^ ^]^ For example, Michal and colleagues focused on the multicentric motion of the knee joint in the sagittal plane, utilizing the multicentricity of a synthetic crossed four‐bar linkage to achieve minimal resistance during motion.^[^ ^20^ ^]^ Although these design approaches can replicate certain actions of the human knee joint, the purely rigid structure results in a significant overall mass and lacks adaptive capabilities when encountering posture changes or unexpected situations, thereby affecting the robot's walking smoothness.

Numerous recent advances in robotics have been inspired by the biological principle of tensegrity and have achieved remarkable feats of dexterity and resilience.^[^ ^21^ ^]^ Tensegrity structures, which combine rigidity and flexibility, offer both rigid support components and a flexible structural system. They can maintain self‐balance and resist impacts when subjected to external forces;^[^ ^22^ ^]^ even a light robot can withstand relatively large impacts and loads,^[^ ^23^ ^]^ and can autonomously return to its initial state depending on its structural configuration. Incorporating this self‐recovery capability into the tensegrity knee joint could avoid the need for a multimotor configuration to achieve knee joint flexion, thereby significantly reducing control complexity and cost. This makes the tensegrity knee joint a solution with strong environmental adaptability and a high stiffness‒to‒mass ratio for robotic systems,^[^ ^24^ ^]^ with promising applications in biomimetic robots,^[^ ^25^ ^]^ such as grippers^[^ ^26^ ^]^ and flexible spherical robots.^[^ ^27^, ^28^ ^]^ Tensegrity structures are very similar to those of biological systems and can be applied to the musculoskeletal systems of organisms.^[^ ^29^, ^30^, ^31^ ^]^ Hao et al. proposed a low‐inertia tensegrity joint protected by a fiber Bragg grating‐embedded silicone sheath.^[^ ^32^ ^]^ Chen et al. proposed a tensegrity‐based robotic fish, in which the rigid parts of the fish's body are connected by tensegrity joints that are designed to be flexible.^[^ ^33^ ^]^ Zappetti et al. utilized the robustness and high flexibility of tensegrity structures to develop an artificial variable‐stiffness spine.^[^ ^34^ ^]^ Kobayashi et al. designed artificial muscles with passive shape adaptability in 3D environments on the basis of tensegrity structures.^[^ ^35^ ^]^ Scarr et al. designed an elbow joint on the basis of tensegrity structures,^[^ ^36^ ^]^ and Li et al. developed a tensegrity hand by designing a specific topology that mimics a human hand.^[^ ^37^ ^]^ Sun et al. explored the similarities between tetrahedral‐mast tensegrity structures and human foot biomechanics and developed an adaptive biomimetic foot mechanism.^[^ ^38^ ^]^ However, the high number of degrees of freedom (DOFs) in tensegrity structures results in a large motion space, making it difficult to match the desired leg movements.^[^ ^39^ ^]^ (Note S4, Supporting Information) To address this, we add a four‐bar mechanism to achieve directional deformation constraints for tensegrity structures, ensuring smooth movement while increasing structural stability.

In this work, we drew inspiration from the human musculoskeletal system and its similarity to tensegrity structures. We simplified the leg musculoskeletal system into a Snelson‐X type tensegrity structure, which was first introduced and demonstrated by American sculptor Kenneth Snelson in the 1940s.^[^ ^40^ ^]^ Our objective is to replicate the dynamic and static behaviors of the human leg by employing tensegrity structure principles. Proceeding from the sliding motion and locking function of biological knee joints, we introduce a four‐bar linkage mechanism that utilizes its singular position to facilitate a rigid–soft transition. However, the four‐bar mechanism does not allow self‐unlocking, leading us to develop an automatic unlocking mechanism based on ropes that can initiate self‐unlocking without external force application; this meets the continuous walking requirement for legged robots. To avoid the increase in cost and control complexity associated with multimotor actuation, we developed a single‐motor‐driven hip joint solution. By utilizing only a single DC motor and a basic Arduino control board, we achieve fluid walking motion. The system's efficacy is validated by constructing test platforms and is supported by data collected through a force platform and a 3D motion capture system; the data confirm the similarity of the system's motion to human gait patterns.

## 2 Results

### 2.1 Design Strategy for Bionic Legged Robot

The human leg plays a crucial role in walking and has an exceedingly complex physiological structure. It comprises not only the tibia and femur, which provide structural support, but also soft tissues such as ligaments, muscles, and tendons. The geometric shapes of the tibial and femoral condyles offer limited constraints, necessitating the collaboration with tendons to harness the tensile forces generated by the contraction and deformation of muscles and ligaments. This rigid–flexible coupling combination approximates a tensegrity structure.

**Figure 1:**
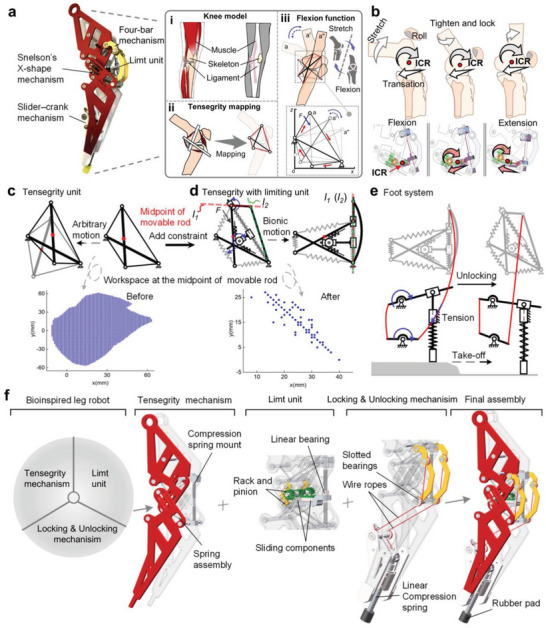
Design strategy for a biomimetic legged robot. a) The design of the robot is inspired by the functional anatomy of the human leg: **a‐i)** knee model; **a‐ii)** tensegrity mapping process; **a‐iii)** flexion function replicated. b) Principle of variable ICR in the knee joint. c) Arbitrary motion of the movable rigid rods within the tensegrity structure and the motion space of the rod midpoints (highlighted in red) before applying directional constraints. d) After directional constraints are applied, the motion of the movable rigid rods changes from a free state to a locked state, and the motion space of the rod midpoints is shown (highlighted in red). e) The unlocking principle of the tendon‐based linkage unlocking mechanism in the mechanical foot system. f) Components of the robot structure. (For the component parameters and assembly procedures, see the Methods section and Note S1, Supporting Information).

Building upon this foundation, we first analyzed the mechanical properties of the skeletal system of the human leg. The tibia and fibula had to be merged into a single bone on the basis of the biological structural features of the human leg, and four dominant muscle bundles were extracted from the leg on the basis of the motion deformation characteristics of the muscular tissue (**Figure** **1**a‐i,ii).^[^ ^41^ ^]^ Bones can be approximated as rigid bodies owing to their high stiffness; by representing them as two rigid rods and considering the coupled relationships of the musculoskeletal system, we simplified them as two rigid members and a continuous elastic network, forming a Snelson‐X type tensegrity structure (Figure 1a‐ii) (for the design parameters and stability analysis, see Notes S2–S4, Supporting Information).^[^ ^40^ ^]^ This structure provides the range of motion (ROM) for the knee joint and equips legged robots with autorecovery capabilities for their knee joints (Figure 1a‐iii).

The core mechanism of knee joint motion concerns the articulating surfaces of the tibia and femur condyles, which result in a combination of sliding and rolling movements on the sagittal plane during flexion and extension of the knee, featuring a variable ICR. Additionally, the knee joint can support substantial loads in an upright position.^[^ ^20^ ^]^ Inspired by the interbone slipping action during the knee‐bending process, we achieve variable ICR functionality by integrating separate rolling and sliding mechanisms (Figure 1b). Specifically, the rolling action is facilitated by the tensegrity units, whereas the sliding action is accomplished through a slider‐track assembly embedded within the structure. This design allows for synchronized rolling and sliding motions with a single external load, eliminating the need for multiple motor drives and complex structural designs.

Furthermore, owing to the multiple redundant DOFs and large ROM provided by the tensegrity units, matching the movement of these units with the leg's movement is challenging (Figure 1c). Inspired by the motion characteristics of biological knee joints, we designed a limiting unit on the structure with two rods and four cables (Note S1, Supporting Information steps 7 to 8), which constrains the redundant DOFs of the mechanism, thereby facilitating the desired motion (Figure 1d). However, this biomimetic unit does not provide locking capability at the end of its travel, meaning that issues such as overflow of the knee joint or insufficient support during the stance phase may occur. Therefore, it is necessary to consider the incorporation of a locking unit into the structure, which must not affect the compliance of the knee joint.

In design, there is often a contradiction between structural stability and compliance. Compliant movement usually requires good structural flexibility, which, however, can reduce the stability of the structure. Similarly, designs that offer high structural stability have high rigidity and an inevitable loss of compliance. Balancing compliance and rigid support is a significant challenge. To address this challenge, we add a locking unit (a four‐bar mechanism) to the tensegrity unit and limiting unit in the knee joint structure. The singular position of the four‐bar mechanism allows locking at the end position of the limiting unit's ROM, effectively emulating the locking of the human leg at full extension during the stance phase. When the joint moves from flexion to extension, the red line *l_1_* and the green line *l_2_* in Figure 1d coincide; therefore, three points in the four‐bar mechanism align on the same plane, and the mechanism enters a locked state.^[^ ^42^, ^43^ ^]^

However, once the mechanism is locked, the knee joint remains extended, preventing continuous walking, which contradicts the design objectives. Inspired by the compliant flexion motion driven by elastic tissues such as ligaments and muscles in the human leg during walking, we design a rope‐based linkage unlocking mechanism (Figure 1e) that mimics the coupling characteristics of leg muscles. This mechanism ensures the automatic unlocking and self‐recovery capabilities of the tensegrity legged robot. The rope transfers the torque produced by the mechanical foot's ejection to the locking mechanism. When the robot's foot is compressed, the rope is in a relaxed state. As the robot's foot leaves the ground, the rope connected to the foot end pulls the robot's locking mechanism to unlock. The elastic potential energy stored in the spring at the knee joint can then assist in the rapid completion of the flexion movement, which is very similar to the function of tendons in the human leg.^[^ ^41^, ^44^ ^]^

### 2.2 Performance Validation of the Bionic Knee Joint

**Figure 2:**
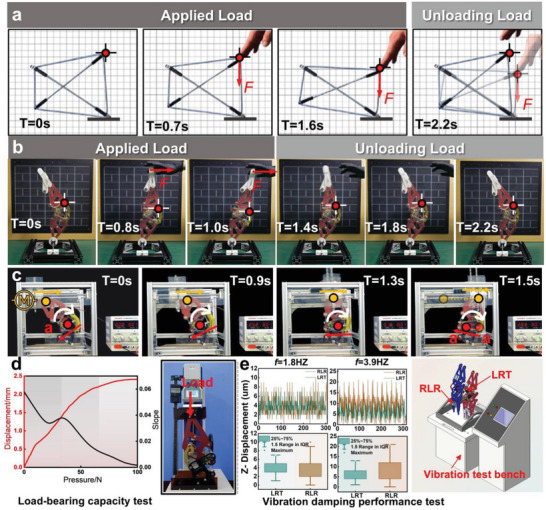
Validation of the performance of the bionic knee joint. a) The Snelson‐X type tensegrity unit demonstrates self‐recovery and self‐stabilization. b) The bionic knee joint exhibits similar self‐recovery and self‐stabilization. c) Compliance in the flexion and extension locking functions is confirmed. d) Load‐bearing capacity of the extension locking of the knee joint. e) Comparison of vibration damping performance between the tensegrity and the rigid (single rotation‐pair) knee joints.

To achieve self‐stabilization and self‐recovery for the bionic knee joint, the concept of a tensegrity structure is incorporated into the design of the knee joint. **Figure** **2**a illustrates the ability of the standard Snelson‐X type tensegrity unit to maintain self‐stability both without an external load and under a single‐point external load. It also demonstrates its adaptability during load application and its self‐recovery performance after load release.^[^ ^45^ ^]^ The bionic knee joint leg structure developed in this paper retains these properties of the independent tensegrity unit (Figure 2b). This is especially evident when the leg‐type robot's knee joint undergoes compliant flexion under artificial loading. Once the load is removed at 1.4 s, the tensegrity unit at the knee joint rapidly returns to its initial state within 0.8 s, confirming its excellent self‐recovery capability. Additionally, the entire experimental process was conducted with the knee joint structure in an inverted position, requiring no external force for stabilization. This further demonstrates its superior structural stability (a theoretical analysis of the structure's stability is provided in Note S2, Supporting Information, and the experimental process is documented in Movie S1, Supporting Information).

After its inherent functions were verified, a test bench was constructed to conduct a validation test of the knee joint compliant flexion‒extension locking function (Figure 2c). The knee joint prototype, driven by a single motor at the hip joint, was smoothly flexed. At 1.3 s, the upper and lower parts of the knee joint approached the self‐locking angle threshold (180°). Within just 0.2 s, the four‐bar mechanism reached the singular position and achieved locking, allowing the knee joint to quickly enter the extension locking phase. The rapid mechanical self‐locking design of the bionic knee joint ensured a high response speed in the transition from compliant to rigid during the extension movement, indicating a rapid and low‐latency transformation process. This fast response mechanism enables the stiffness of the knee joint to be quickly adjusted to increase the robot's support capability (the experimental process is shown in Movie S2, Supporting Information).

The load‐bearing capacity in the extended locking state was subsequently tested (Figure 2d). The lower end of the extended and locked knee joint was fixed to the test platform, and the hip joint was connected to a digital dynamometer. A vertical downward force ranging from 0 to 100 N was applied. Initially, manual loading of the four‐bar mechanism to the singular point position was required. When the knee joint, which stores elastic potential energy, was mounted on the test platform and loaded, some elastomers still had to recover from their bent and displaced positions during the loading process. Therefore, the displacement change in this process was the fastest (slope of 0.06). In the second stage, owing to assembly clearances within the robot, the knee joint was in the gap‐filling phase, during which the slope gradually decreased (from 0.04 to 0.02). In the third stage, the curve was relatively flat, with a slope close to 0 (the slope at 100 N was only 0.0014). At this point, the four‐bar structure could be considered to have achieved locking, meaning that the robot met the rigid support requirements. To avoid structural damage from excessive loading, further loading was halted.

Additionally, the knee joint designed in this paper is a rigid–flexible coupling unit, unlike the traditional single revolute joint knee structure. To explore the vibration damping potential of this new type of knee joint structure during walking (1.8 Hz) and running (3.9 Hz) movements,^[^ ^46^ ^]^ we conducted comparative experiments with traditional revolute joint knees and tensegrity knees on a vibration platform. Specifically, under the same vibration source, both joints were completely fixed to the vibration platform, and sensors were placed above the knee joints to measure the transmission of vibrations through the knee joint structure, as shown in Figure 2e. The interquartile range was defined as 25%–75%, and the interquartile range (IQR) method was used to exclude outliers. By recording the amplitudes, it was found that under the same vibration source input conditions, the tensegrity knee joint showed a 29% reduction in vibration displacement during walking and a 38% reduction during running compared with the traditional revolute‐joint knee. This effectively demonstrated its vibration damping function (the experimental process is documented in Movie S3, Supporting Information).

### 2.3 Application Potential in Walking Motion

The bionic knee joint demonstrates a harmonious integration of adaptability and high‐load support in robotic applications. By exploiting the singular position characteristic of a four‐bar linkage, the intrinsically compliant and less rigid tensegrity knee joint swiftly transitions into a high‐stiffness locked structure when in an upright position (a high‐load condition), thereby inhibiting bending and providing substantial support capability. However, the inherent limitation of the singular position of the four‐bar mechanism prevents unlocking under continuously increasing loads, as confirmed by the experiments shown in Figure 2d. Hence, the development of an automatic unlocking mechanism is fundamental for simplifying the control system and facilitating locomotion.

**Figure 3:**
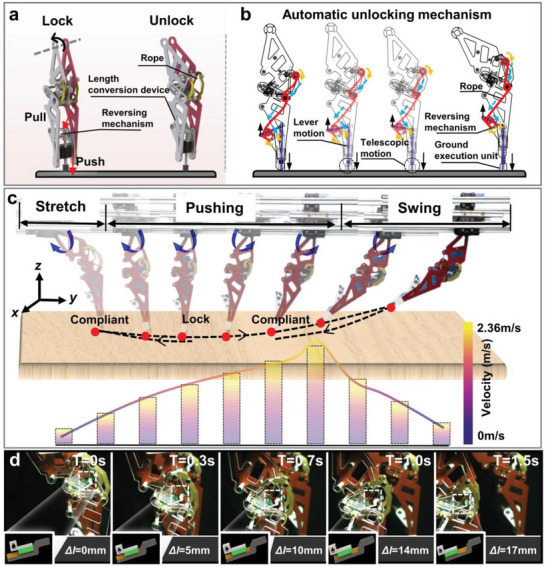
Functional validation of the legged robot in walking mode**. a)** Rope‐based automatic unlocking mechanism. b) Sketch of the automatic unlocking mechanism. c) The compliant–rigid–compliant continuity walking test of the legged robot is assisted by spring deformation restoration within the unlocking mechanism, with peak velocities at the moment of pushing off from the ground. d) Calibration of the knee joint microslip during locomotion (variable ICR).

In this design, we leverage the high compliance and rapid response of a rope‐driven approach to develop an automatic rope‐based unlocking mechanism (**Figure** **3**a).^[^ ^47^, ^48^ ^]^ This mechanism consists of a system rope, a ground execution unit, and a reversing mechanism. The rope, similar to leg muscles, connects various modules to form a closed‐chain structure. The ground execution unit, a crank–slider mechanism, is divided into an extendable foot and a length conversion device. The length conversion device's output end connects to the reversing mechanism via a short rope, transforming the foot's extension motion into a pulling force on the knee joint locking mechanism. This is facilitated by the rotation of a conversion rod around an axis connected to the robot's lower leg shell, which performs a lever motion.

Building on the unlocking mechanism design, elastic elements are used to increase the robot's response speed, with ropes attached to elastic joints resembling the robotic musculoskeletal system proposed by Li et al.^[^ ^37^ ^]^ This unlocking mechanism operates in two states: energy storage and energy release. In the energy storage state, the four‐bar mechanism on either side of the knee joint is locked in the energy storage state, and the longitudinal compression of the extendable foot mechanism causes the connected rope to slacken. Conversely, in the energy release state, the vertical popping out of the extendable foot tightens the rope, swiftly moving the four‐bar mechanism away from the singular position and thereby unlocking it (Figure 3b). Thus, a coupling mechanism for knee and foot locking–unlocking is designed (Movie S4, Supporting Information).

In the single‐leg continuous walking test, a test platform design that restricted motion in the *x*‐direction while allowing motion in the *y‐* and *z‐*directions was used. A DC motor located at the hip joint drove the thigh clockwise to touch the mechanical foot down, gradually transitioning the legged robot from a flexed to an extended state. Upon reaching the upright extended state, the four‐bar mechanism entered the singular position, achieving a transition from compliance to rigidity. As the robot pushed off, the ground actuation unit was propelled by the spring within the automatic unlocking mechanism, pulling the rope downward and disengaging the four‐bar mechanism from the singular position. This transition from rigidity back to compliance allowed for continuous walking functionality (Figure 3c). Empowered by the assistive force from spring deformation recovery, the legged robot disengaged the knee joint lock and completed a single gait cycle within 274 ms; this high‐speed response capability is also demonstrated in Figure 2c (Movie S5, Supporting Information).

Additionally, a high‐speed camera (i‐SPEEDTR) was employed with the test platform to calibrate the minute slippage at the robot's knee joint (Figure 3d). During knee flexion, this movement can be decomposed into rotation and sliding motions. The rotation is facilitated by the compliant deformation of the tensegrity unit and a limiting unit, whereas the sliding is provided by a sliding pair acting as the human patella. The displacement of the slider is directly proportional to the state transition time, reaching a maximum displacement of 17 mm when the knee joint is locked. This replicates the minor slippage movement of the human knee joint, thereby achieving its variable ICR functionality (Movie S6, Supporting Information).

**Figure 4:**
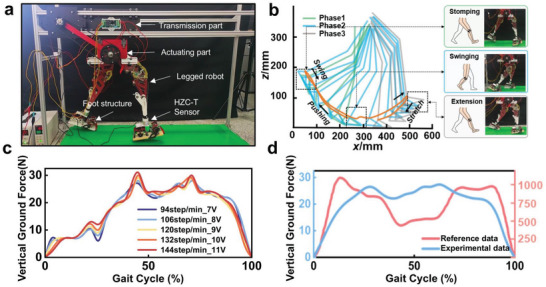
Verification of humanoid gait consistency. a) Walking experiment environment of the legged robot. b) Legged robot foot trajectory, which can be divided into three stages, namely, the stomping stage, swinging stage, and extension stage, compared with a human gait on the walking platform. c) Variation in the ground reaction force (GRF) of the legged robot at different frequencies. d) Graph comparing the legged robot's GRF with the human body's normal walking GRF under the same gait cycle (the data in the literature are derived from reference^[^ ^51^ ^]^).

To further increase the objectivity of the experiment and to better replicate human walking actions and gait, we replaced the original ground actuation unit with a previously studied anthropomorphic foot structure^[^ ^43^, ^49^ ^]^ (Note S5, Supporting Information). We conducted a walking experiment with the robot on a stationary platform (**Figure** **4**a). Drawing on the analysis of foot motion trajectories in different scenarios by Mendez,^[^ ^50^ ^]^ we selected a congruent gait cycle and used a 3D capture system to extract the motion trajectory of the robot's foot end (Figure 4b). The closed orange curve in the figure indicates the fluidity and uniformity of the robot's walking gait.

By adjusting the pulse width modulation values at the input, we altered the experimental step frequency to measure the impacts of different step frequencies on the ground reaction forces (GRFs) of the legged robot depicted in Figure 4c. In the initial phase of gait (0–10%), groups with lower step frequencies were more responsive in terms of force. Heel contact with the ground occurred during the 20–30% phase of the gait cycle; at this point, the groups with higher step frequencies had a greater increase in slope, indicating a positive correlation between the velocity at heel strike and the GRF. As the gait reached the 30–50% phase, the foot rolled on the ground, the knee joint locked, and the curve reached its first peak. The peak data revealed that the 144‐steps‐per‐minute group experienced a 14% increase in force compared to that of the 94‐steps‐per‐minute group, illustrating the relationship between step frequency and GRF (Movie S7, Supporting Information).

As the heel lifted off the ground and the angle at the toe joint with the ground increased, the toe joint executed a push‐off when the knee joint unlocked, leading the curve to its second peak. The increase for the 144‐steps‐per‐minute group at this second peak was only 7% relative to the force of the 94‐steps‐per‐minute group, which was less than the increase at the first peak. This is because the average velocity during the stance phase, when the foot is in contact with the ground, is less than that during the swing phase. The differences in the push‐off speeds between the groups were minimal. This confirms that the legged robot has walking characteristics similar to those of humans (Figure 4d).

**Figure 5:**
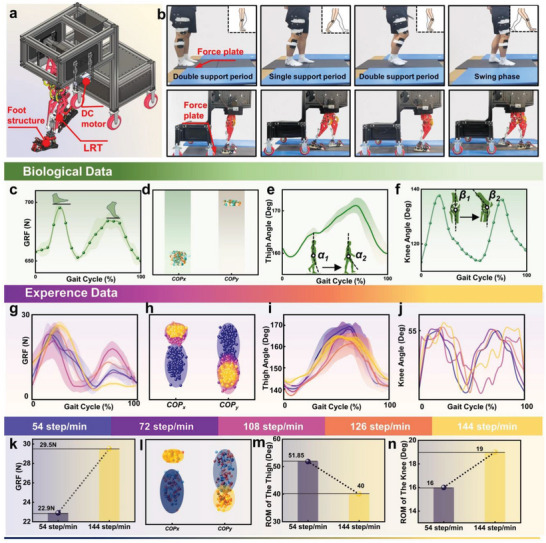
Functional validation on an open bipedal walking platform. a) Structural composition of the open walking platform. b) Comparison between the natural human gait and the gait of the legged robot. c–f) Measured changes in the GRF, centre of pressure (COP), thigh angle, and knee angle of the human body during a single gait cycle. g–j) GRF, COP, thigh angle, and knee angle data for a single gait cycle of the legged robot at multiple stride frequencies (with speeds increasing in steps from the blue region to the yellow region). k–n) Differential analysis of the GRF, COP, thigh angle, and knee angle for legged robots in high (144 step min^−1^) and low (54 step min^−1^) stride frequency conditions.

The abovementioned fixed bipedal walking platform could confirm the high consistency between the legged robot's gait and human walking patterns, yet it was limited in terms of walking distance. To further assess the functional performance of the proposed system, we developed an experimental platform that integrated the multi terrain adaptability of the legged robot with the carrying ability of a vehicle (**Figure** **5**a). Additionally, under the same measurement conditions, we collected standard human gait data. The experimental process and the results of human data collection are shown in Figure 5b,c–f, respectively.

During the robot's walking process, we collected data on the robot's GRF, center of pressure (COP), thigh joint angle, and knee joint angle under multiple step‐frequency conditions (Figure 5g–j). The GRF curves exhibited a typical double‐peak pattern (Figure 5g), which was a direct reflection of the forces produced when the heel strikes and the toe leaves the ground while walking. With increasing step frequency, the higher‐step‐frequency groups experienced a greater increase in peak force in the GRF curves than did the lower‐step‐frequency groups. Specifically, at higher step frequencies, the peak force values increased by an average of 28% compared with the forces at lower step frequencies (Figure 5k). To analyze the walking stability of the legged robot, we investigated the distribution of the COP during walking. As shown in Figure 5h, the COP data points are mainly concentrated in a smaller area, indicating that the pressure points changed little during the walking process. With increasing step frequency, the robot improved the response speed and balance efficiency by narrowing the range of the pressure center,^[^ ^52^ ^]^ thus achieving dynamic stability during walking (Figure 5l). Furthermore, we analyzed the changes in the legged robot's thigh angle throughout the gait cycle (Figure 5i). Under different step frequencies, the trend of changes in the robot's thigh angle showed a high degree of consistency. As the step frequency of the robot gradually increased, a smaller stride was needed to adapt to shorter gait cycles; that is, the ROM of the thigh angle decreased from 51.85° to 40° (Figure 5m). To verify the adaptability of the knee joint angle of the legged robot with a stretched overall structure under different step frequencies, we collected knee joint angles for five different step frequencies. Figure 5n shows that at a step speed of 54 steps min^−1^, the knee joint angle was 53°, whereas at 144 steps min^−1^, the knee joint angle increased to 56°. This demonstrates that the legged robot needed to increase the bending angle of the knee joint to adapt to a faster step speed, which in turn accelerated the swinging speed of the leg and reduced the gait cycle (Movie S8, Supporting Information). Additionally, walking tests under various road conditions (including uphill, downhill, grass, sand, and cobblestone paths) were conducted. The results closely approximated the trends observed in the human data. For detailed information, please refer to Note S8 (Supporting Information).

## 3 Conclusion

In this paper, a bionic legged robot is developed, inspired by the human leg; the robot is based on a tensegrity structure designed by extracting and simplifying the biological musculoskeletal system via bionic design principles. The robot can complete a full gait cycle for a single leg using only a single motor and a basic control program. The rigid–flexible coupling structure significantly reduces the weight; a single leg weighs only 925 g, which is much lighter than those of other rigid‐leg robots (Table S7 and Figure S9, Supporting Information). Inspired by the variable ICR of the human knee, a separate rolling and sliding mechanism was introduced to replicate this action. It was proven to reduce the amplitude vibration displacement by 29% and 38% under normal walking (1.8 Hz) and running (3.9 Hz) frequencies, respectively, thereby mitigating the adverse effects of vibrations transmitted through the knee joint. A four‐bar mechanism was arranged on the lateral side of the knee, utilizing its singular point characteristics to achieve unified free flexion and extension locking. A vertical force gauge was used to apply a 100 N load, resulting in only a minor 1.04% deformation, confirming the stability of the locking module. An automatic unlocking mechanism based on ropes was developed, allowing the legged robot to switch from compliance to locking during the flat‐foot phase, providing upright support. It also switches from locking to compliance at the moment of pushing off (426 ms), achieving smooth flexion. These abilities are expected to increase the range of its applications in the field of bipedal robots and support applications in lower‐limb rehabilitation research, providing new ideas for the development of new lower‐limb rehabilitation equipment.

However, our current design is still at the conceptual prototype stage, and significant work remains to be done before it can be applied to humanoid robots and wearable technologies. We acknowledge the limitations of the current materials and the complexity of the assembly process, such as the gap errors caused by numerous separate parts during the assembly process—as evidenced by the rapid rise of the curve in Figure 2d—and the relatively low stiffness of the parts made from polylactic acid (PLA) material. Therefore, in subsequent research, we will integrate the separate parts and use uniform rigid materials to increase the structural stiffness, enabling the mechanism to carry greater weight and supporting its expansion to humanoid robots and wearable applications.

## 4 Experimental Section

### Physical Prototype Fabrication

The dimensions of the legged robot with tensegrity (LRT) are designed on the basis of one‐third of the normal human body parameters. Taking a healthy individual with a height of 180 cm as an example, the length of the thigh is ≈620 mm, and the length of the calf is ≈494 mm. Consequently, the total length of the robot's thigh is 207 mm, and the total length of the robot's calf is 165 mm. The physical prototype fabrication of the bionic legged robot involves the creation of a main body with rigid outer shells on both sides (Figure 1e). One side is made from aluminum alloy material, and a laser cutter is used to cut the main structure of the mechanical leg from a prefabricated 5052 aluminum alloy plate (5 mm thick), followed by sandblasting and anodizing. To display the internal joint movements of the legged robot, the other side is cut from a 5 mm thick acrylic plate to create an identical structure. The connection components of the robot's outer shell consist of eight precisely ground 304 stainless steel light shafts embedded in acrylonitrile butadiene styrene plastic bushings. Considering the assembly process, one spring that bears a compressive load in the Snelson‐X shape tensegrity structure was replaced with two springs that bear tensile loads, resulting in six linear springs (diameter 0.5 mm) connected to the light shafts. Another compression spring uses a 5 mm diameter light shaft as an installation rod, positioned between two #45 steel connectors, with the upper connector serving as the main component for transmitting torque; a linear bearing (LM5UU) is used to eliminate friction during movement, thus forming a stable tensegrity structure.

Inside the robot's two outer shells, two racks (1 module) are installed to mesh with standard gears (1 module). The central shafts of the two standard gears are fixed to the slider of a miniature linear rail (MGN 7C) through connectors (#45 steel). The rail that mates with the slider is installed on a #45 steel rail connector, which is equipped with a linear bearing (LM5UU) in the middle to mimic the function of the patella in biology; this bearing is integrated with the compression components on the compression spring installation rod, forming the limit unit of the mechanical knee joint module that enables variable‐ICR movement. Another purpose of the bionic knee joint module is to provide rigid support, for which an engineering solution consisting of a mechanical four‐bar linkage locking unit was explored. The locking mechanism is composed of two sets of laser‐cut long and short rods (5052 aluminum alloy plate, 3 mm thick) and two cylindrical pins (#45 steel quenched) for singular point limitation. The long and short rods are articulated, with the short rods connected to the thigh part of the robot's outer shell and the long rods connected to the calf part; all articulated positions are equipped with miniature deep groove ball bearings. The cylindrical pins are symmetrically installed at the singular points on both sides of the robot's thigh, ensuring the reliability of the locking structure.

The automatic unlocking mechanism consists of a 3D‐printed body part and a reversing mechanism (Polymax PLA 1.75 mm, X1E, Bambu Lab), 304 stainless steel light shafts, a linear bearing (NSK LM10UU), grooved bearings (V1804, CCH), a linear spring (φ0.9 mm), and a steel wire (φ1 mm, DEKDEJA company). A set of grooved bearings is installed at the articulated positions of the long and short rods to directly pull the locking mechanism away from the singular point to unlock it; another set of grooved bearings is located on the robot's calf connecting rod to perform tensioning. (For the detailed installation procedures for the legged robot, see Note S1, Supporting Information)

### Functional Validation of the Knee Joint—Self‐Recovery Performance

The constructed Snelson‐X type tensegrity unit consists of two identical‐length 304 stainless steel shafts (φ3 mm) and four tension springs of the same wire diameter (φ0.9 mm). The legged robot was inverted and mounted on a fixed frame to maintain structural stability (Figure 2a,b). Both the tensegrity unit and the legged robot were manually loaded and subsequently released, resulting in nearly identical adaptive deformation and self‐recovery behaviors.

### Functional Validation of the Knee Joint—Compliance in Flexion‒Extension Locking

1. Isolated Testing: The knee joint was separated from the legged robot and installed on a test bench composed of a square profile frame (European Standard 2020) and linear bearing‒shaft assembly, powered by a regulated power supply that delivered 8 V to a single DC motor, which executed the movement from a highly flexed to a fully extended position.
2. In vivo testing: An aluminum profile frame was placed on the floor, with the robot's initial position marked to ensure that each experiment started from the same location. A slide rail was mounted at the top of the frame to provide freedom in the *y*‐direction, with a single DC motor installed at the hip joint of the robot to drive the legged robot's extension movement. The drive motor and the robot's hip joint were mounted on the aluminum frame using 8 mm thick acrylic plates and 3D‐printed connectors, enabling the robot to move along the horizontal slide rail. The drive motor was manually controlled via a wireless controller, which allowed the robot's hip joint to begin the next cycle at a new position upon completion of a motion cycle.

### Functional Validation of the Knee Joint—Vibration Damping Performance

In this experiment, a comparative study was conducted on the vibration transmission characteristics of rigid‐legged robots and legged robots with tensegrity. A 6‐DOF electromagnetic vibration platform was used to simulate the impact of ground vibration on the legs of the robots while walking and running. The legs of both types of robots were fixed to the electromagnetic vibration platform. Referring to the frequency data for human walking and running from the literature,^[^ ^46^ ^]^ vertical vibrations were applied at frequencies of 1.8 and 3.9 Hz, respectively. The vibration intensity was set to 100%, and the waveform was a sine wave. Wireless Bluetooth vibration sensors (WTVB01‐BT50, Wit‐motion Co., Ltd.) were installed above the knee joints of both legged robots to record the transmission of vibrations in real‐time. The vibration platform applied vibrations according to the set frequency and intensity while the sensors collected vibration displacement data. After data collection, the raw data were preliminarily filtered via signal processing software, and outliers were excluded via the IQR method. Then, the filtered data were analyzed to calculate the mean and standard deviation of the vibration displacement to obtain more reliable results.

### Functional Validation of the Knee Joint—Load‐Bearing Capacity

To test the support capability of the legged robot in a standing locked position, the robot's lower leg was fixed to the testing platform in the erect locked state. The robot's hip joint was connected to an SF‐100 N digital force gauge (Aipli Co., Ltd), and a vertical load of 0–100 N was applied by manually turning the feed knob. The displacement during the loading process was recorded by a vertical digital scale installed on the testing platform.

### Gait Potential Experiment

To simulate human walking movements and gait, an experimental protocol was developed in which independent motors were used to drive the hip joints. The previously studied anthropomorphic foot structure^[^ ^43^, ^49^ ^]^ was replaced with the original ground‐engaging unit, and an HZC‐T tension‒compression sensor was installed at the ankle joint to measure the mechanical performance of the legged robot upon ground contact. The experimental setup, as shown in Figure S7 (Supporting Information), consisted of a 2‐DOF actuation platform and transmission linkage. The cooperation between the slide rail and trolley provided the legged robot with freedom in the *y* and *z* directions during operation. Additionally, the parallelism constraint ensured a low friction coefficient and smooth movement during sliding. The transmission linkage provided torque through a motor, with a 1:2 gear ratio belt drive delivering stable torque in periodic clockwise and counterclockwise directions. The flexibility of the transmission process could mitigate the impact during walking and allow vibrations caused by uncontrollable factors to be absorbed, ensuring that the lower limb structure could smoothly complete various movement postures and maintain the fluency of the gait cycle (the test bench design scheme is detailed in Note S5, Supporting Information). To assess the functional performance of the legged robot, a Qualisys 3D motion capture system was deployed in the walking experiment area to obtain real‐time motion data on the robot's gait.

To further validate the walking performance of the legged robot, an experimental platform was developed that integrates the multi terrain adaptability of the legged robot and the vehicle carrying capacity (Figure 5a), and walking trials were conducted with both a human subject and the legged robot. During the walking trials, a Qualisys Miqus M3 motion capture system (Qualisys Co. Ltd.), in conjunction with a BERTEC FP4060‐05‐PT‐2000 force plate (BERTEC Co. Ltd.), was used to measure the GRF, COP, thigh angle, and knee angle of the human and the legged robot under five different step frequencies (54, 72, 108, 126, 144 steps min^−1^). The legged robot achieved human‐like gait movements with only a single 12 V, 16 RPM DC motor driving the hip joint. The basic control program was compiled into executable binary files on an Arduino development board via the Arduino IDE compiler on a PC and then uploaded to the board. The program invoked an L298 N motor driver module to drive the DC gear motor, and a Bluetooth module connected to the Arduino development board via RX and TX ports enabled speed control of the DC gear motor through signals sent from a mobile device's Bluetooth serial port. The control configuration and wiring diagram are detailed in Note S6 (Supporting Information).

## Conflict of Interest

The authors declare no conflict of interest.

## Supporting information



Supporting Information

Supplemental Movie 1

Supplemental Movie 2

Supplemental Movie 3

Supplemental Movie 4

Supplemental Movie 5

Supplemental Movie 6

Supplemental Movie 7

Supplemental Movie 8

## Data Availability

The data that support the findings of this study are available from the corresponding author upon reasonable request.
