# Supplementary material for: Legged Robot with Tensegrity Feature Bionic Knee Joint
Source: Adv Sci (Weinh). 2025 Feb 3;12(12):2411351. doi: 10.1002/advs.202411351 (PMC11948042; doi:10.1002/advs.202411351)
Supplement: Supplementary file 1 — Supporting Information [file ADVS-12-2411351-s004.pdf]

ADVANCED SCIENCE
Open Access

Supporting Information

for *Adv. Sci.*, DOI 10.1002/advs.202411351

Legged Robot with Tensegrity Feature Bionic Knee Joint

Qi Wen, Meiling Zhang, Jianwei Sun, Weijia Li, Jinkui Chu, Zhenyu Wang, Songyu Zhang and Luquan Ren*

Supporting Information

Legged robot with tensegrity feature bionic knee joint

Authors:

Qi Wen[1], Meiling Zhang[1*], Jianwei Sun[1], Weijia Li[1], Jinkui Chu[2], Zhenyu Wang[2], Songyu Zhang[1], and Luquan Ren[3]

Affiliations:

[1]School of Mechatronic Engineering, Changchun University of Technology, China.

[2]School of Mechanical Engineering, Dalian University of Technology, China.

[3]Key Laboratory of Bionic Engineering, Jilin University, China.

[*]*Meiling Zhang* Email: Zhangml@hrbeu.edu.cn.

The PDF file includes:

Supplementary Note S1 to S9

Other Supporting Information includes the following:

Movie S1 to S8

Note S1 Assembly drawing

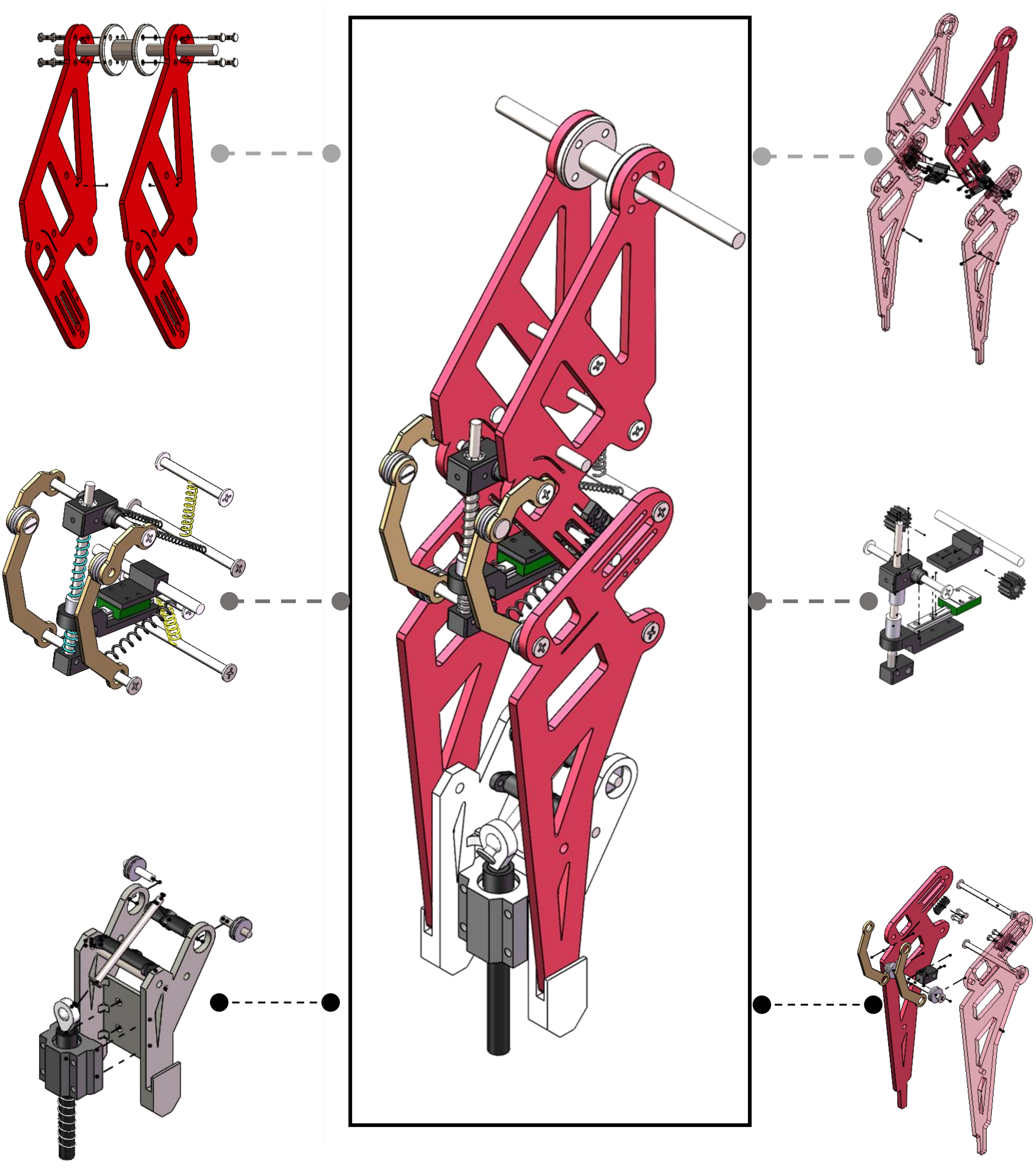

Legged Robot

PROTOTYPE

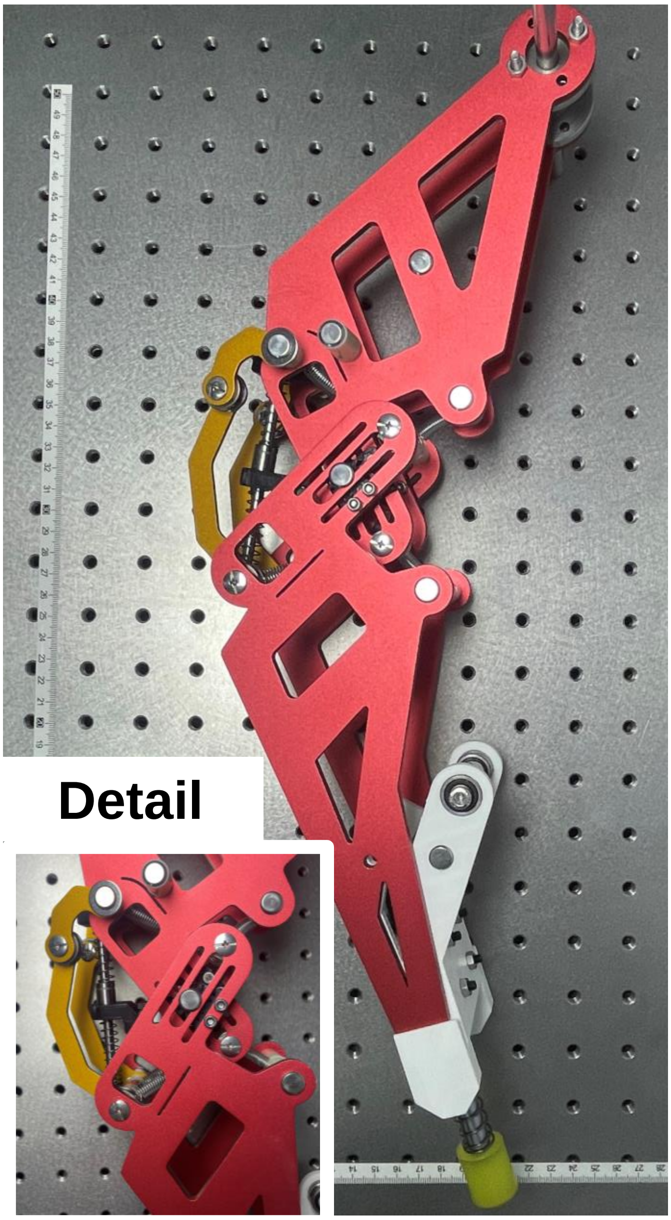

Stretch

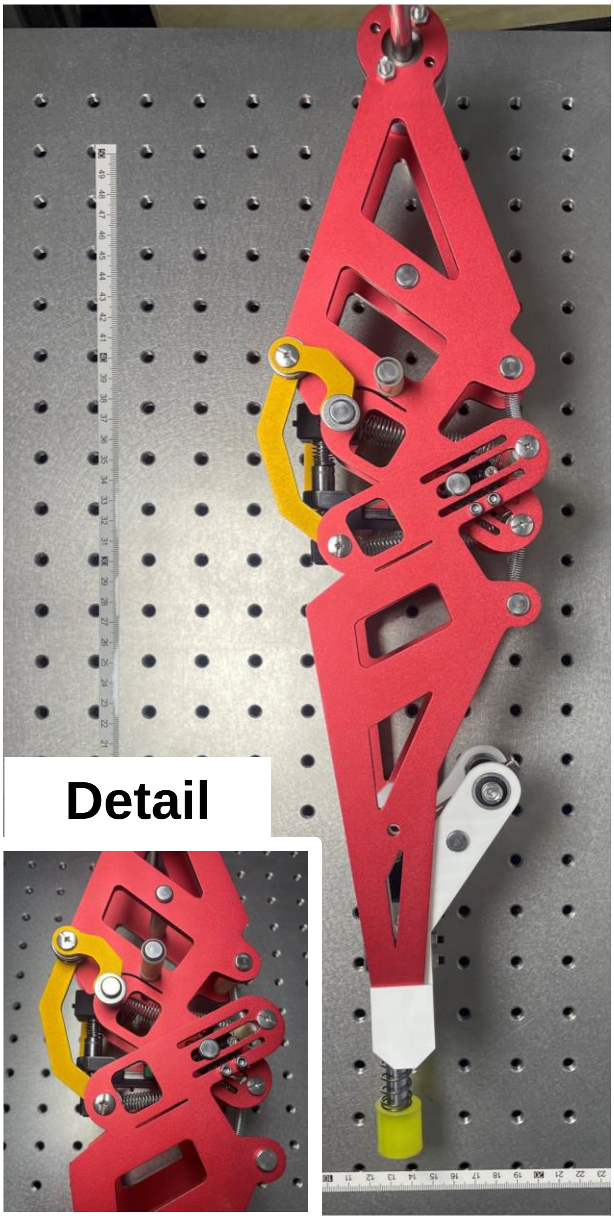

Locking

Legged Robot

PHYSICAL PICTURE

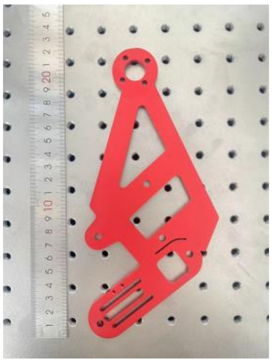

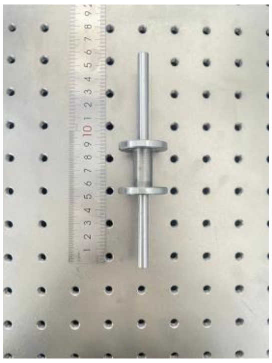

Thigh link **Stepped shaft**

3D MODEL

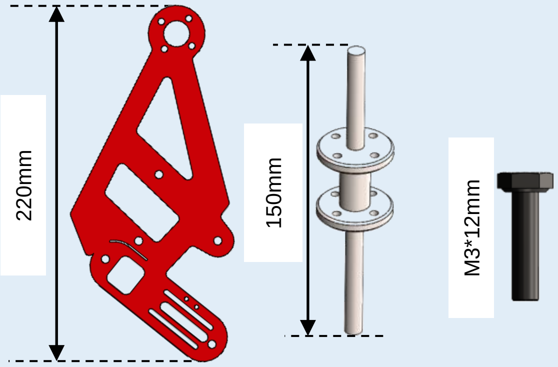

1

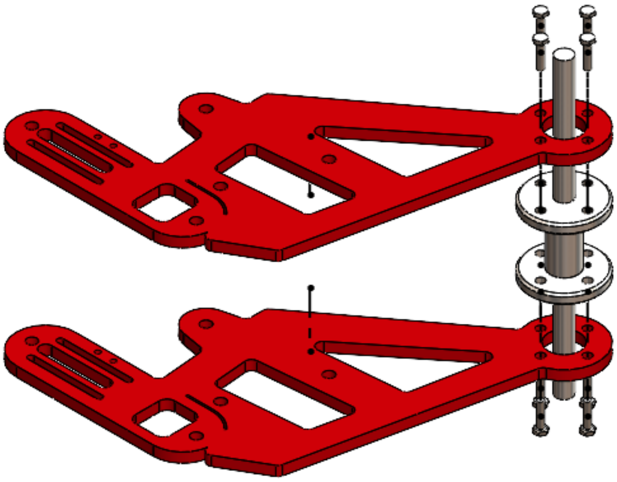

PHYSICAL PICTURE

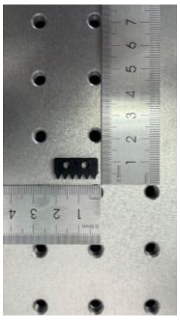

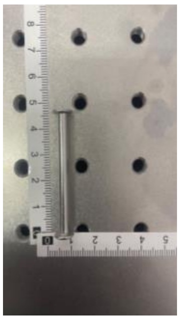

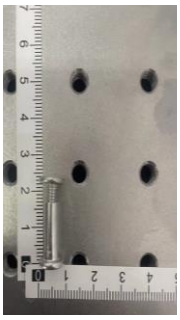

Pinion **WZBS**

3D MODEL

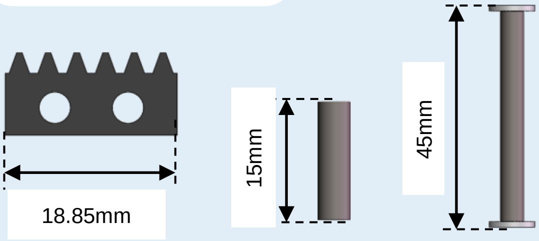

2

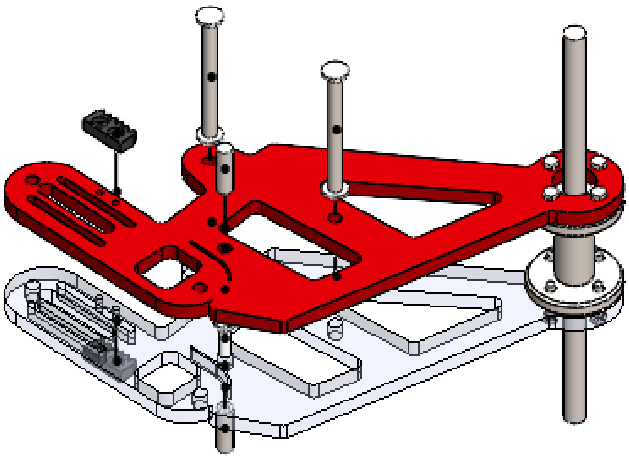

PHYSICAL PICTURE

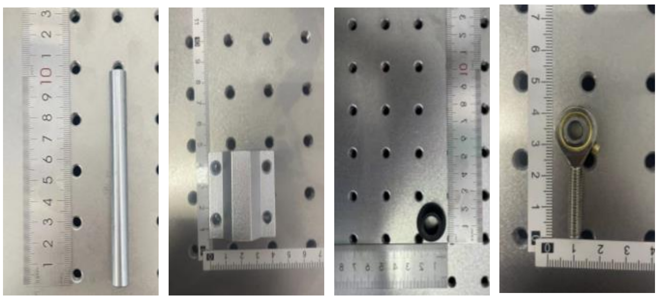

Steel shaft | Straight bearing seat | 3D printed parts (PLA) | Bearing

3D MODEL

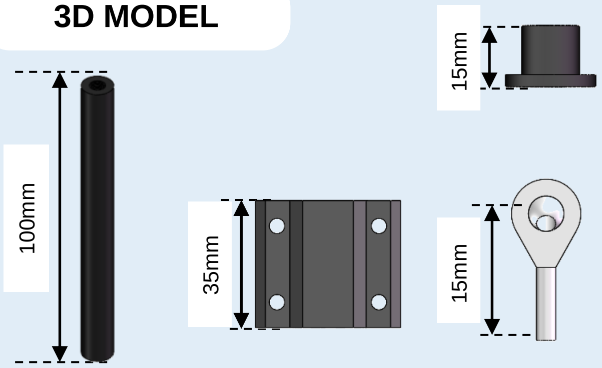

3

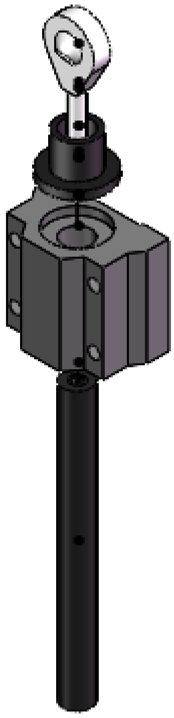

PHYSICAL PICTURE

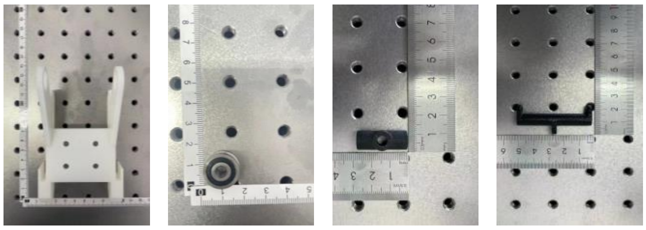

3D printed parts (PLA) | Bearing | 3D printed parts (PLA) | 3D printed parts (PLA)

3D MODEL

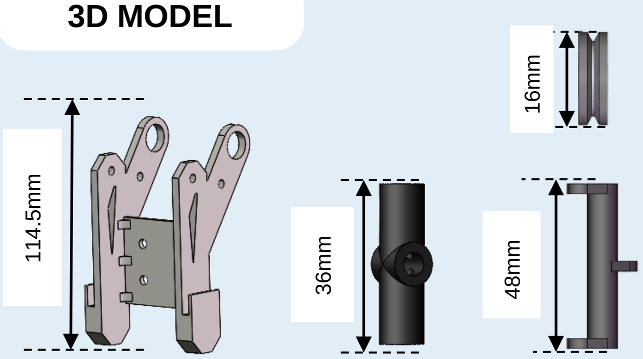

4

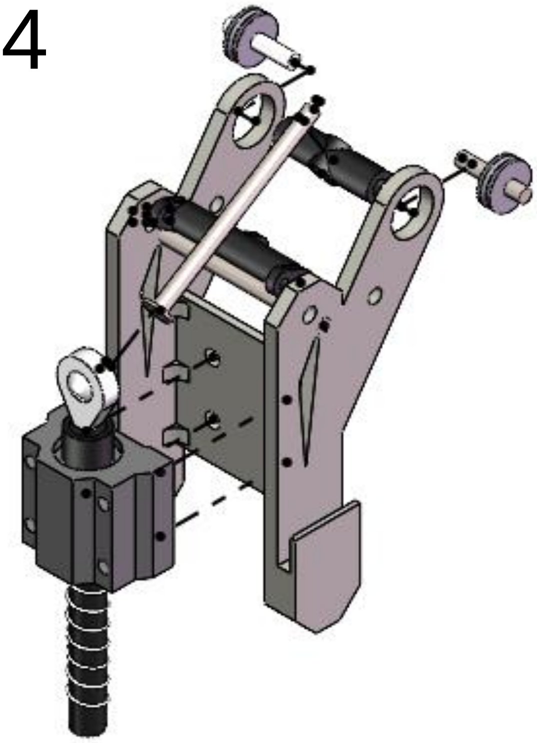

PHYSICAL PICTURE

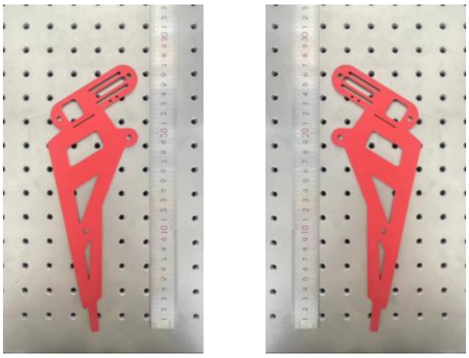

Shank link

3D MODEL

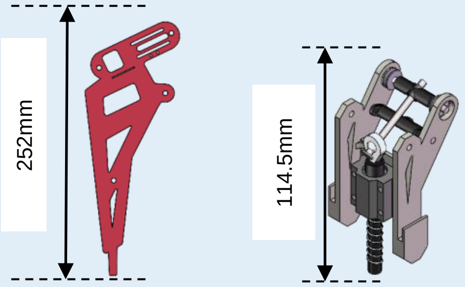

5

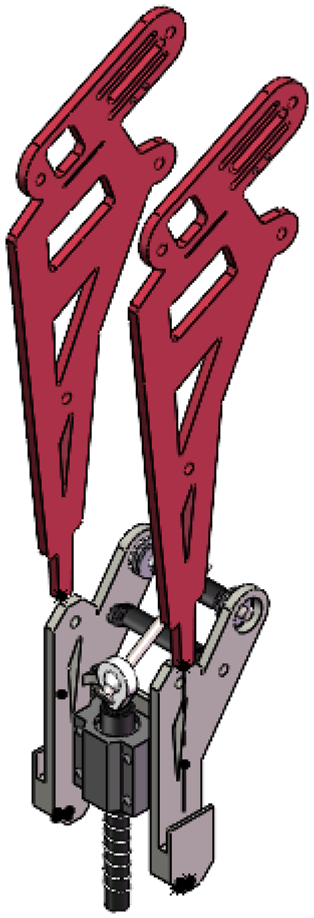

PHYSICAL PICTURE

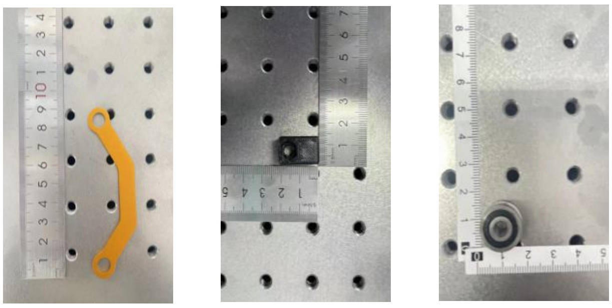

Locking long link

3D printed parts (PLA)

Bearing

3D MODEL

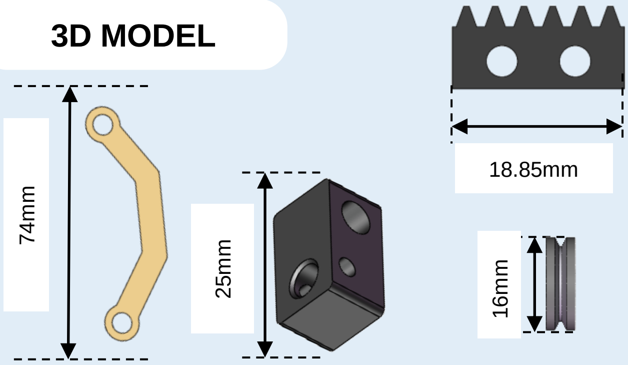

6

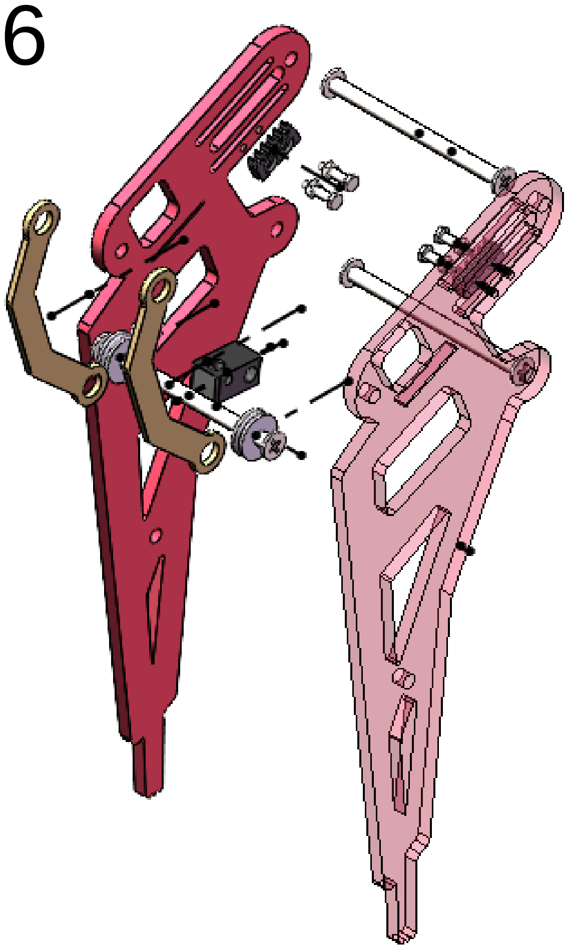

7

PHYSICAL PICTURE

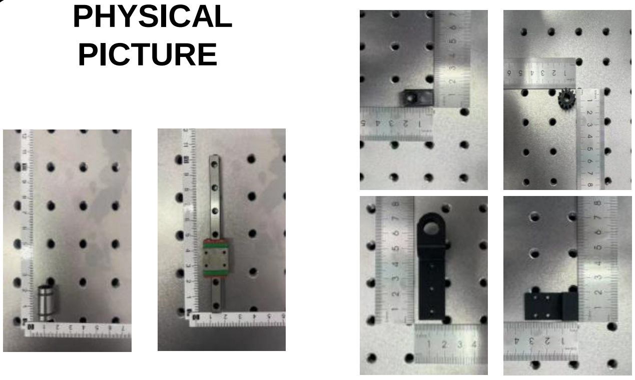

Linear bearing | **MGN7C Slider** | **3D printed parts (PLA)**

3D MODEL

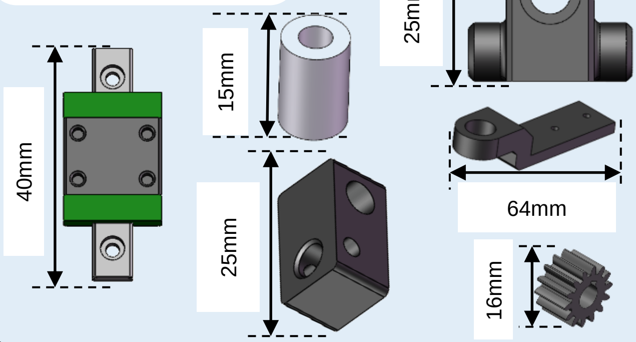

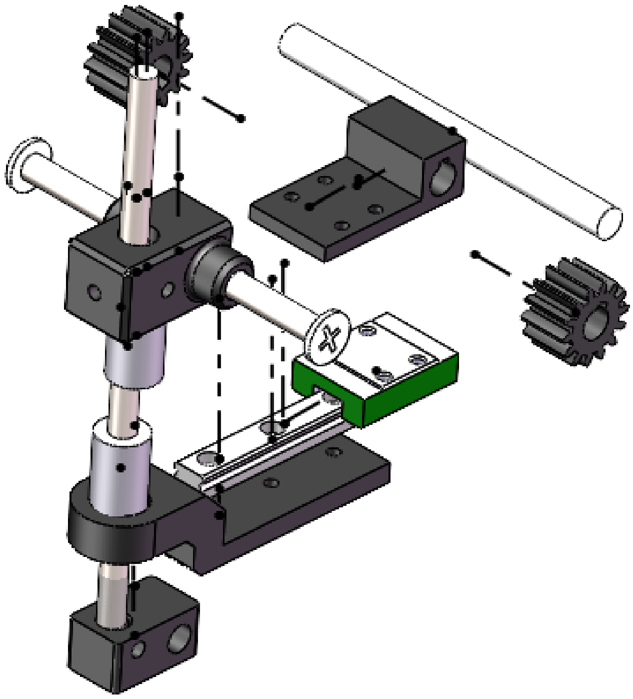

8

PHYSICAL PICTURE

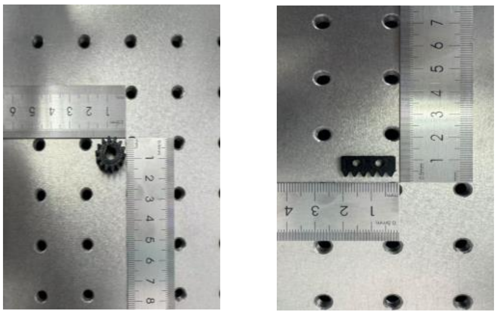

Pinion and Rack

3D MODEL

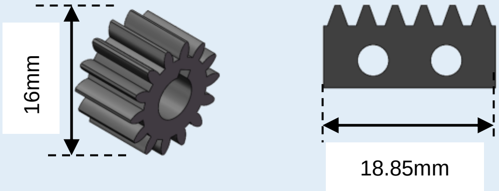

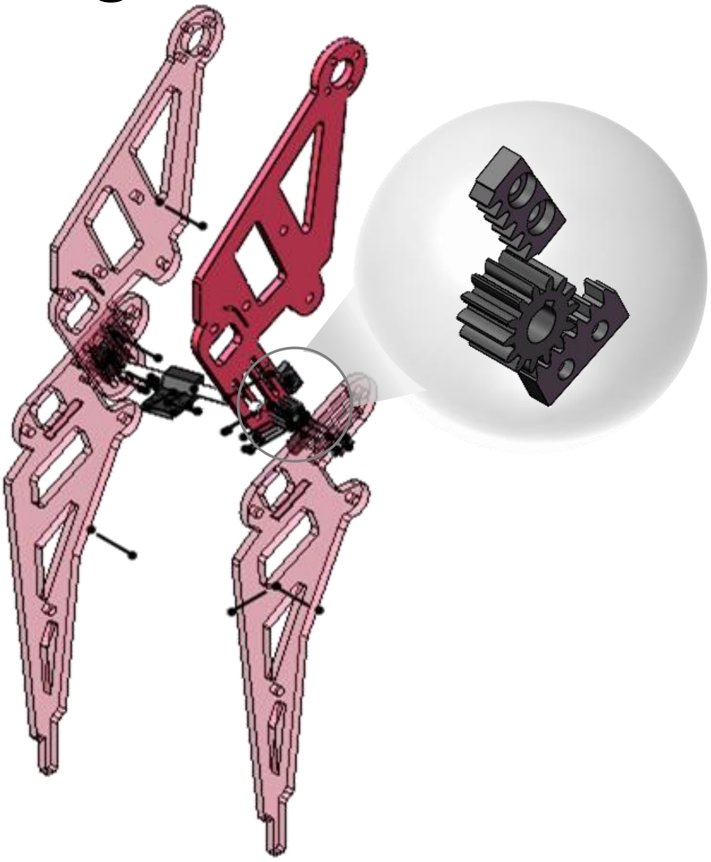

Locking Unit

PHYSICAL PICTURE

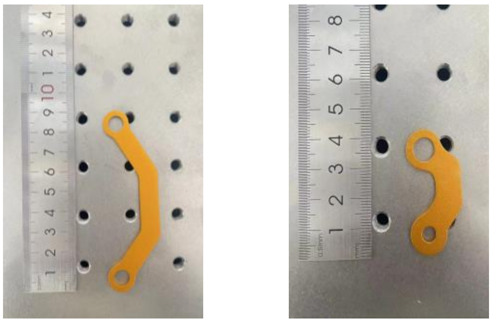

Pinion and Rack

3D MODEL

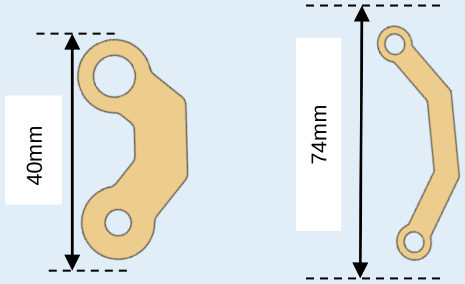

9

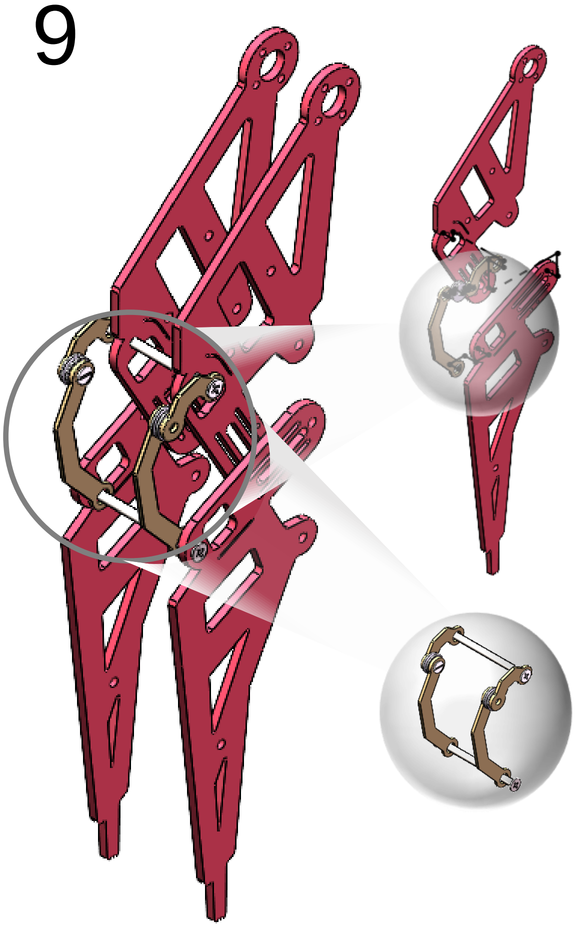

PHYSICAL PICTURE

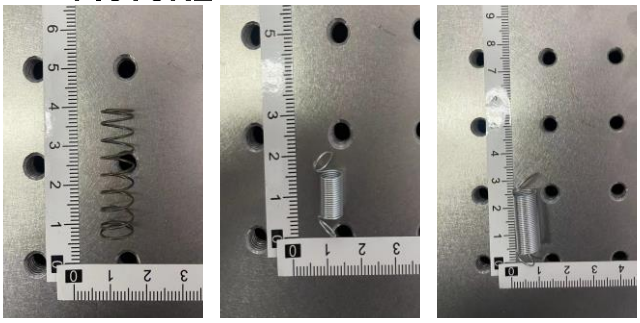

Pressure spring

Tension spring

3D MODEL

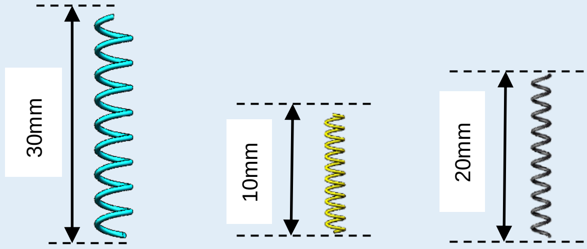

10

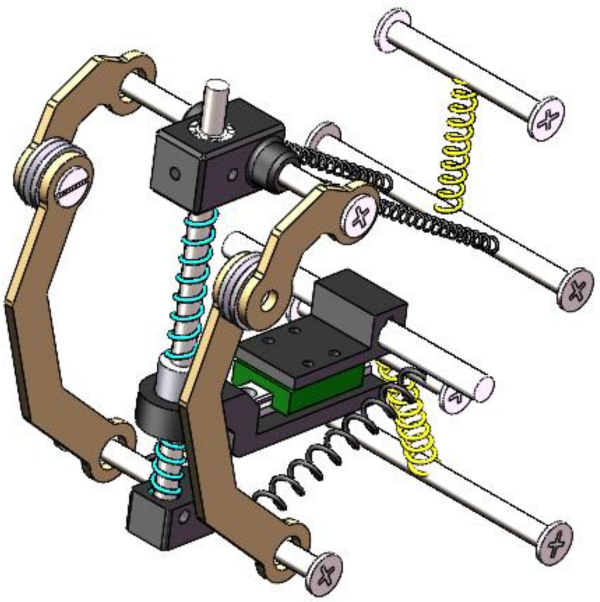

- **Note S2 Stability analysis of the tensegrity structure.**

It is known that the mechanism unit of a two-bar and four-cable plane tensegrity structure includes rigid and elastic members, and the nodes defining the rigid and elastic members are denoted by *p*. The robot has 6 mechanism units and 4 nodes, and its topological connection relationship can be represented by the connection matrix *C*.

Defining the two end node numbers of the mechanism unit as *i* and *j* ($i < j$), the connectivity matrix *C* can have the following equation:

$$C = \begin{pmatrix} 1, & p = i \\ -1, & p = j \\ 0, & \text{Remaining situation} \end{pmatrix} \tag{1}$$

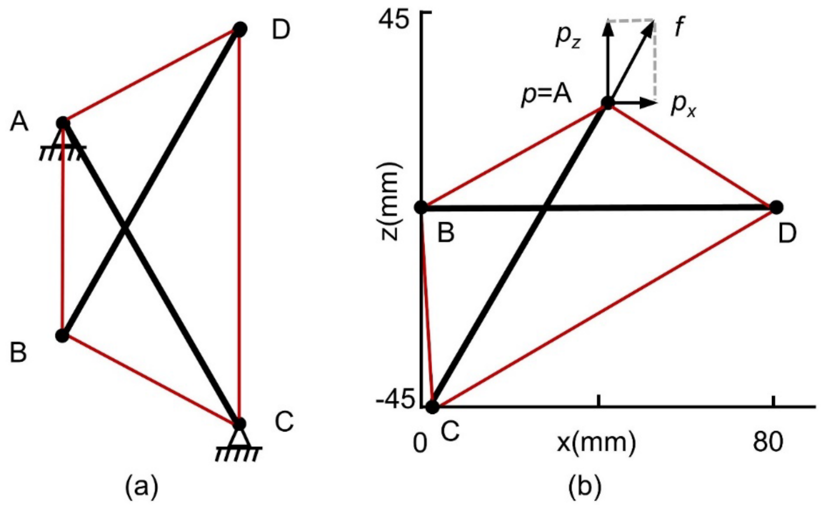

Figure S1. Mechanism schematics and coordinate drawings.

Define the four nodes of the two-bar and four-cable plane tensegrity structure as A, B, C, and D. The mechanism principle of the robot is shown in Figure S1(a). Therefore, the six mechanism units are AB, AD, CD, BC, AC, and BD, where the mechanism units AB, AD, CD, and BC are elastic members and the mechanism units AC and BD are rigid members.

The connection matrix for this robot mechanism is created using Eq.(1), as shown in Table S1.

Table S1 Organizational unit node connection matrix

Institutional unit	Matrix C			
	A	B	C	D
AB	1	-1	0	0
AD	1	0	0	-1
CD	0	0	1	-1
BC	0	1	-1	0
AC	1	0	-1	0
BD	0	1	0	-1

The connection matrix of the robot mechanism is obtained by collation:

$$C=\begin{bmatrix} 1 & -1 & 0 & 0 \\ 1 & 0 & 0 & -1 \\ 0 & 0 & 1 & -1 \\ 0 & 1 & -1 & 0 \\ 1 & 0 & -1 & 0 \\ 0 & 1 & 0 & -1 \end{bmatrix} \tag{2}$$

On the two-bar and four-cable plane tensegrity structure, the coordinate system $O\text{-}xz$ shown in Figure S1(b) is established, and the ratio of the axial force of the mechanism unit to the length of the member is defined as the coefficient of the force density q. The diagonal matrix Q, composed of the force density q of the member, can be expressed as follows.

$$Q = diag(q) \tag{3}$$

The equilibrium equations were developed:

$$C^T QCx = p_x \tag{4}$$

$$C^T QCz = p_z \tag{5}$$

Where x, z are column vectors consisting of the coordinates of the four nodes of the two-bar and four-cable plane tensegrity structure in the coordinate system $O\text{-}xz$. p_x,p_z are column vectors consisting of the components of the external force acting on node p in the x and z directions, respectively.

Define the force density matrix $D = C^T QC$ This can be obtained by combining Eq.(2) and (3):

$$D = \begin{bmatrix} q_1 + q_2 + q_5 & -q_1 & -q_5 & -q_2 \\ -q_1 & q_1 + q_4 + q_6 & -q_4 & -q_6 \\ -q_5 & -q_4 & q_3 + q_4 + q_5 & -q_3 \\ -q_2 & -q_6 & -q_3 & q_2 + q_3 + q_6 \end{bmatrix} \quad (6)$$

Using the coordinate matrix $[x\ z]$ of the node p of the mechanism unit, Eqs.(4) and (5) can be simplified as:

$$C^T QC[x\ z] = D[x\ z] = [P_x\ P_z] \quad (7)$$

Define the length of the mechanism cell as l_{ij} (i=1, 2, 3, j=1, 2, 3, 4; $i < j$). Then, the length of each mechanism an be expressed respectively as AB=l_{12}, AD=l_{14}, CD=l_{34}, BC=l_{23}, AC=l_{13}, BD=l_{24}.

In order to comply with the actual physiological parameters of the human body, the mechanical knee joint dimensions are defined according to the dimensional parameters of the biological knee joint. The length of rigid members AC and BD is taken as 80 mm. The length of the elastic members AD and BC is taken as 45 mm. Determining the dimensional parameters of the tensioning integral mechanism unit in conjunction with the angular range of leg flexion and extension in the human walking motion. As shown in Table S2.

Table S2 Mechanism unit size parameters

Element	l_{12}	l_{14}	l_{34}	l_{23}	l_{13}	l_{24}
Lengths/mm	48.5	45	90	45	80	80

According to the coordinate diagram of the mechanism shown in Figure 1(b), the coordinates of the nodes of the mechanism unit in the coordinate system *O-xz* are obtained using the dimensional parameters of each mechanism unit in Table S2, as shown in Table S3.

Table S3 Organizational unit node coordinates

	A	B	C	D
x	42	0	2	80
z	24.25	0	-45	0

The properties of the two-bar and four-cable plane tensegrity structure itself can be obtained:

$$D[x\ z]=[0\ 0] \tag{8}$$

Combining Table S3 and Eq.(6), substitute into q_1=1, getting q_2=0.538, q_3=0.29, q_4=0.539, q_5=-0.539, q_6=-0.538. The diagonal matrix Q can be expressed as：

$$Q = diag\left(q\right) = diag\left(1, 0.538, 0.29, 0.539, -0.539, -0.538\right) \tag{9}$$

It can be observed: $q_1, q_2, q_3, q_4 > 0$, $q_5, q_6 < 0$. Satisfying the relationship between the geometric configuration of the tensegrity structure and the structural parameters.

When the mechanism is in equilibrium, it can be expressed as:

$$Aq = \left[P_x\ P_z\right] \tag{10}$$

where matrix A is the equilibrium matrix of the organization

$$A = \begin{bmatrix} C^T diag(Cx) \\ C^T diag(Cz) \end{bmatrix} \tag{11}$$

Substituting the data from Table S3 into Eq.(11) yields the node balance matrix of the body unit:

$$A = \begin{bmatrix} 42 & -38 & 0 & 0 & 40 & 0 \\ -42 & 0 & 0 & -2 & 0 & -80 \\ 0 & 0 & -78 & 2 & -40 & 0 \\ 0 & 38 & 78 & 0 & 0 & 80 \\ 24.25 & 24.25 & 0 & 0 & 69.25 & 0 \\ -24.25 & 0 & 0 & 45 & 0 & 0 \\ 0 & 0 & -45 & -45 & -69.25 & 0 \\ 0 & -24.25 & 45 & 0 & 0 & 0 \end{bmatrix} \tag{12}$$

Elastic stiffness matrix of the mechanism K^E.

$$K^E = \left(AL^{-1}\right) G \left(AL^{-1}\right)^T \tag{13}$$

which L=diag(l_{12},l_{14},l_{34},l_{23},l_{13},l_{24}). G is the diagonal matrix consisting of the axial stiffness deformations of the mechanism units. The axial stiffness deformations of the elastic members are the force density coefficients q, and the axial stiffness deformations of the rigid members are approximated by 0.

Therefore, $L = diag\left(48.5, 45, 90, 45, 80, 80\right)$, $G = diag\left(1, 0.538, 0.29, 0.539, 0, 0\right)$

The eigenvalues of K^E can be obtained:

$$eig(K^E) = \left(2.2105, 0.4441, 1.0799, 1.0058, 0, 0, 0, 0\right) \tag{14}$$

Therefore the matrix K^E has quadratic form $Q\left(K^E\right) > 0$, K^E is positive definite matrix.

According to the theory of prestress stability, the two-bar and four-cable plane tensegrity structure is stable, i.e. its stability does not depend on prestressing and material properties, and the geometric stiffness matrix K^G of the mechanism is semi-positive definite.

Tangential stiffness matrix K:

$$K = K^E + K^G \tag{15}$$

Consequently $Q\left(K\right) = Q\left(K^E\right) + Q\left(K^G\right) > 0$, the tangent stiffness matrix K is a positive definite matrix.It can be judged that the two-bar and four-cable plane tensegrity structure is stable under prestressing.

- **Note S3 Kinematic analysis and spring stiffness matching of a robot with a tensegrity structure.**

The stiffness of elastic members in tensegrity structure can affect the overall stiffness of the mechanism. The stiffness of each elastic member of the two-bar and four-cable plane tensegrity mechanism is established by the inverse dynamics method.

Establish the coordinate system for the motion of the mechanism, and the spatial positions of the mechanism nodes p = A, B, C, and D are shown in Figure S2 (a).

The mechanism node p will make a small translational motion along the z-axis while making a rotational motion around the coordinate system *O-xyz*, and the chi-square coordinates of the motion p'

$$p' = Rp + V \tag{16}$$

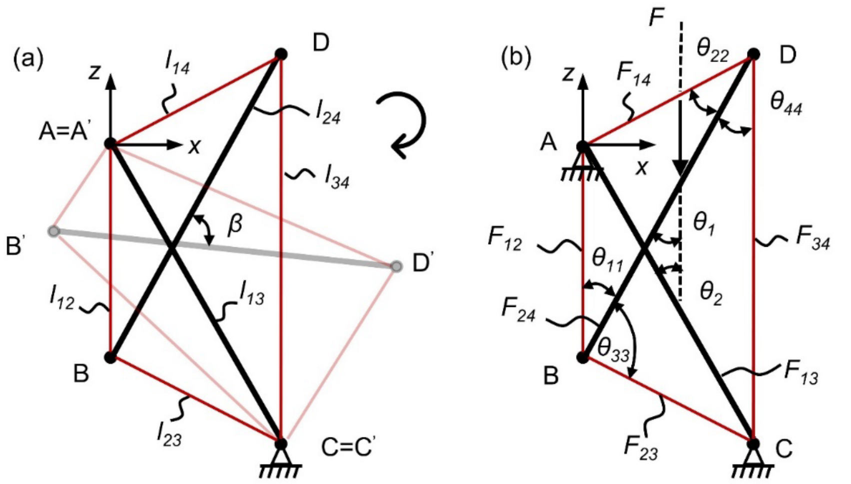

Figure S2. Schematic diagram of force on mechanism

$V=[0\quad 0\quad v]^T$ is the translation distance of the member along the *z*-axis and *R* is the rotation matrix.

$$R=\begin{bmatrix} c\alpha c\beta & -s\alpha c\gamma + c\alpha s\beta s\gamma & s\alpha s\gamma + c\alpha s\beta c\gamma \\ s\alpha c\beta & c\alpha c\gamma + s\alpha s\beta s\gamma & -c\alpha s\gamma + s\alpha s\beta c\gamma \\ -s\beta & c\beta s\gamma & c\beta c\gamma \end{bmatrix} \tag{17}$$

To simplify the matrix, *cos* is denoted by *c* and *sin* is denoted by *s*. The matrix is expressed as follows where γ is the angle of rotation of the member around the *x*-axis, β is the angle of rotation of the member around the *y*-axis, and α is the angle of rotation of the member around the *z*-axis.

In the coordinate system *O-xyz,* the nodes $p' = A', B', C', D'$ after the movement of the mechanism units. The deformation of each elastic member of the mechanism is:

$$\delta_{ij} = l_{ij}^{'} - l_{ij} \tag{18}$$

Due to the mechanical similarity between human leg muscles and springs, linear springs are chosen to replace the elastic members in the two-bar and four-cable plane tensegrity structure. Matching the stiffness of the spring unit can provide a reference for the selection of the spring member when the legged robot prototype is fabricated, which facilitates the construction of the robot's target pose, and then debugging the pre-stress needed for the robot's adaptive motion within a limited number of spring morphology variables.

Define the stresses in each institutional unit in the stable state of the two-bar, four-cable tensegrity structure as F_{ij} (i=1, 2, 3, j=1, 2, 3, 4, $i < j$), as shown in Figure S2(b).

The nodal force balance equation of the mechanism is:

$$\sum F_x = 0 \tag{19}$$

$$\sum F_z = F \tag{20}$$

Based on the symmetry of the rigid rods of the tensegrity structure during movement, combining Eq. (17) and Eq. (18), the stress Fij of each mechanism unit can be obtained as follows:

$$\begin{bmatrix} \sin\theta_1 & -\sin\theta_2 \\ \cos\theta_1 & \cos\theta_2 \end{bmatrix} \begin{bmatrix} F_{24} \\ F_{13} \end{bmatrix} = \begin{bmatrix} 0 \\ F_z \end{bmatrix} \tag{21}$$

$$\begin{bmatrix} \sin\theta_{11} & -\sin\theta_{33} \\ \cos\theta_{11} & \cos\theta_{33} \end{bmatrix} \begin{bmatrix} F_{12} \\ F_{23} \end{bmatrix} = \begin{bmatrix} 0 \\ F_{24} \end{bmatrix} \tag{22}$$

$$\begin{bmatrix} \sin\theta_{22} & -\sin\theta_{44} \\ \cos\theta_{22} & \cos\theta_{44} \end{bmatrix} \begin{bmatrix} F_{14} \\ F_{34} \end{bmatrix} = \begin{bmatrix} 0 \\ F_{24} \end{bmatrix} \tag{23}$$

Stiffness of the spring unit K_{ij}

$$K_{ij} = F_{ij} I \delta_{ij} \tag{24}$$

Considering the range of angles required by the legs during the actual walking process of the human body, the angular range of flexion and extension motions of the legged robot is designed to be $0° - 56°$, i.e. the angle of rotation of the movable rigid rod of the tensegrity structure around the y-axis $\beta = \left(0° - 56°\right)$. Since the legged robot design only considers its motion in the sagittal plane, both α and γ are 0°, and the shape variable δ_{ij} of the elastic member during the motion of the mechanism can be derived from Eq. (16).

According to the dimensional parameters of each mechanism unit and their positional relationship in the coordinate system, the angular parameters of each mechanism unit can be obtained as shown in Table S4:

Table S4 Mechanism angle parameters

θ_1	θ_2	θ_{11}	θ_{22}	θ_{33}	θ_{44}
30°	30°	30°	32.47°	87.53°	30°

The external force on a legged robot can be determined by its weight. Based on the above relationship, the stiffness of each spring unit can be solved using matlab when the external knee force of the legged robot is certain.

Take for example the force F=245N applied to the outer knee joint of the robot, i.e:

$$\sum F_z = 245\text{N} \tag{25}$$

Substituting the mechanism angle parameters from Table S5 into equations (19), (20) and (21), the stresses in each mechanism unit can be obtained as shown in Table S5.

Table S5 Mechanical unit stress

	F_{13}	F_{24}	F_{12}	F_{14}	F_{23}	F_{34}
Stress/N	141.450	141.450	159.364	79.756	79.756	85.636

Therefore the stiffness of each spring unit in the two-bar and four-cable plane tensegrity structure is shown in Table S6:

Table S6 Spring unit stiffness

	K_{12}	K_{14}	K_{23}	K_{34}
Stiffness/(N/mm)	10.653	4.142	4.142	1.784

● Note S4 Spatial analysis of tensegrity structure unit motion

The more degrees of freedom of the tensegrity structure will allow for a large range of movement space, making it difficult to match the leg movements. Based on the controllability of the tensegrity structure, the redundant degrees of freedom of the two-bar and four-cable plane tensegrity mechanism can be constrained. Combining the motion mechanism of the biological knee joint and the motion characteristics of the bionic legged robot, the target motion is determined to be the symmetric motion of the rigid members of the tensegrity structure.

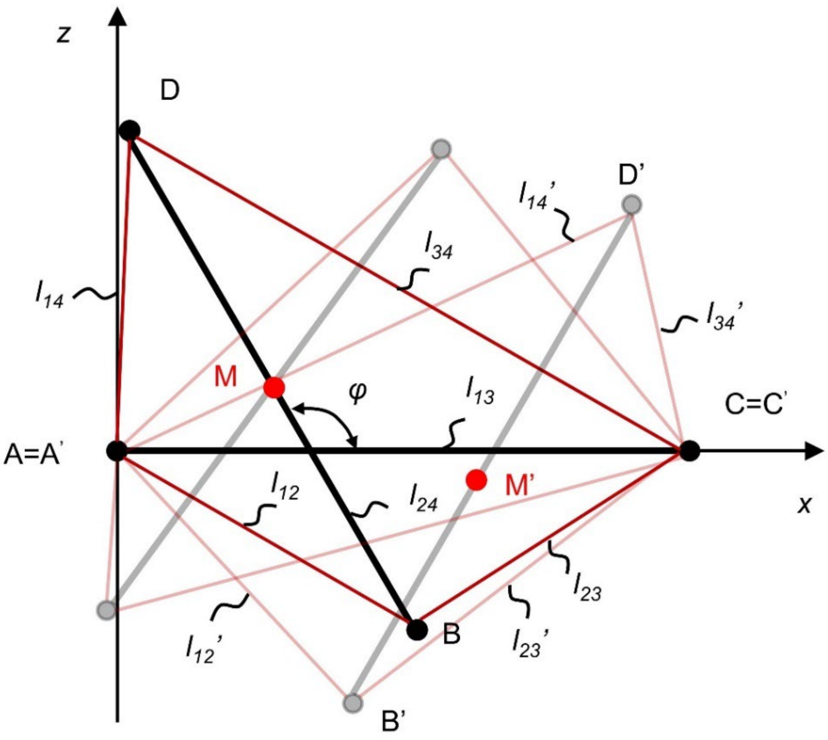

Figure S3. Arbitrary movement of the tensegrity structure.

According to the motion characteristics of the tensegrity structure, numerical methods are used to solve the workspace of the two-bar and four-cable plane tensegrity mechanism. Define the coordinates of the organization node as

$A=(A_x,A_z)$, $B=(B_x,B_z)$, $C=(C_x,C_z)$, $D=(D_x,D_z)$. Original length of elastic member $|AB|=l_{12}$, $|BC|=l_{23}$, $|CD|=l_{34}$, $|AD|=l_{14}$. Rigid member length $|AC|=l_{13}$, $|BD|=l_{24}$. As shown in Figure S3. The coordinates of the nodes after the movement of the mechanism unit are $A'=\left(A_x',A_z'\right)$, $B'=\left(B_x',B_z'\right)$, $C'=\left(C_x',C_z'\right)$, $D'=\left(D_x',D_z'\right)$. The length of the rigid member is constant during the movement of the mechanism, where AC is a fixed rigid linkage and BD is a movable rigid linkage.

Define the length of the elastic member after motion as l_{ij}' (i=1,2,3;j=1,2,3,4;i<j), thus the lengths of the elastic members in the post-movement mechanism can be expressed respectively as: $|A'B'|=l_{12}'$, $|B'C'|=l_{23}'$, $|C'D'|=l_{34}'$, $|A'D'|=l_{14}'$.

According to the determined geometrical parameters of the two-bar, four-cable tensegrity structure, the coordinates of the agency nodes A and C in the coordinate system shown in Figure S3 can be obtained as $A=(0,0)$, $C=(80,0)$.

The midpoint of the movable rigid bar is selected as the designated reference point to analyze the geometric workspace of the tensegrity structure. Define the midpoint of a movable rigid bar $M=(M_x,M_z)$, the angle between rigid member AC and rigid member BD is φ. The connection of each rigid and elastic member in the tensegrity structure is articulated, due to the limitation of its working range $\varphi\in[0°, 180°]$.

Based on the positional relationship of the members, the coordinates of the mechanism nodes B and D can be introduced.

$$\begin{cases} B_x = M_x - \cos\varphi \times l_{24}/2 \\ B_z = M_z - \sin\varphi \times l_{24}/2 \end{cases} \tag{26}$$

$$\begin{cases} D_x = M_x + \cos\varphi \times l_{24}/2 \\ D_z = M_z + \sin\varphi \times l_{24}/2 \end{cases} \tag{27}$$

$$l_{12}' = \left[\left(A_x - B_x\right)^2 + \left(A_z - B_z\right)^2 \right]^{1/2} \tag{28}$$

$$l_{23}' = \left[\left(B_x - C_x\right)^2 + \left(B_z - C_z\right)^2 \right]^{1/2} \tag{29}$$

$$l_{34}' = \left[\left(C_x - D_x\right)^2 + \left(C_z - D_z\right)^2 \right]^{1/2} \tag{30}$$

$$l_{14}' = \left[\left(A_x - D_x\right)^2 + \left(A_z - D_z\right)^2 \right]^{1/2} \tag{31}$$

Since the length change of each elastic member under the action of external force is finite, under the condition of satisfying Hooke's law, each elastic member should satisfy

$$l_{\min} \le l_{ij}' \le l_{\max} \tag{32}$$

where l_{min} and l_{max} are the minimum and maximum lengths allowed for elastic members in a two-bar, four-cable tensegrity structure, respectively.

Selection of the length of the elastic member according to the dimensional parameters of the mechanism unit $l_{12}'\in[18,79]$, $l_{23}'\in[15,75]$, $l_{34}'\in[60,120]$, $l_{14}'\in[15,75]$. Define the midpoint of a movable rigid bar $M_x\in[-100,100]$, $M_z\in[-100,100]$. The step size of

the loop variable is $\Delta x = \Delta y = 1$, $\Delta\varphi = \pi / 180$ .The workspace of the two-bar, four-cord tensegrity structure with specified reference points is obtained as shown in Figure S4.

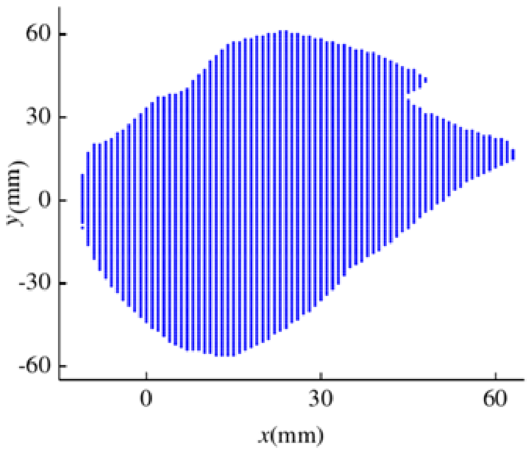

Figure S4. Tensegrity structure primitive motion space.

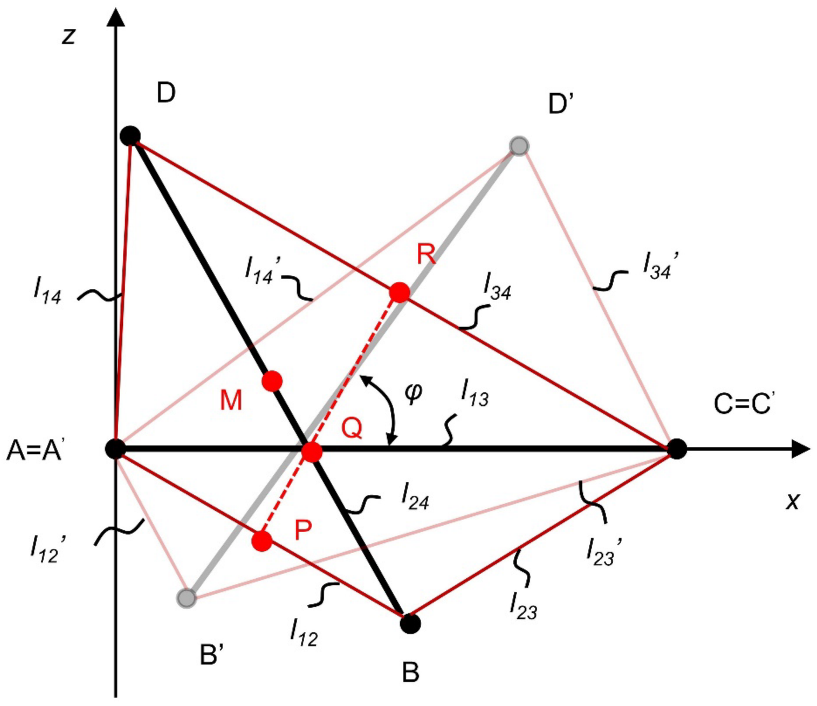

Figure S5. Target motion of the tensegrity structure.

It can be seen that the tensegrity structure has a large range of motion in the workspace, which is caused by its redundant degrees of freedom. Therefore, it is necessary to constrain the symmetry of the rigid rod motion of the tensegrity structure.

Define the midpoint of the elastic member AB as $P=\left(P_x,P_Z\right)$ the midpoint of CD is $R=\left(R_x,R_Z\right)$.

$$\begin{cases} P_x=\left(A_x+B_x\right)/2 \\ P_z=\left(A_z+B_z\right)/2 \end{cases} \tag{33}$$

$$\begin{cases} R_x=\left(C_x+D_x\right)/2 \\ R_z=\left(C_z+D_z\right)/2 \end{cases} \tag{34}$$

Define the intersection of rigid members AC and BC to be $Q=\left(Q_x,Q_z\right)$, thus:

$$(Q_z-B_z)/(Q_x-B_x)=(D_z-B_z)/(D_x-B_x) \tag{35}$$

which $Q_z=0$, Eq. (35) can be changed to:

$$Q_x=B_x-B_z\times\left(D_x-B_x\right)/\left(D_z-B_z\right) \tag{36}$$

Eventually, this can be expressed as:

$$(Q_z-P_z)/(Q_x-P_x)=(R_z-Q_z)/(R_x-Q_x) \tag{37}$$

Thus, the workspace of the midpoint M of the movable rigid bar of the two-bar, four-cable tensegrity structure after adding constraints is obtained as shown in Figure S6. Symmetry constraints can effectively limit the redundant degrees of freedom of the tensegrity structure and realize the basic motion of the legged robot.

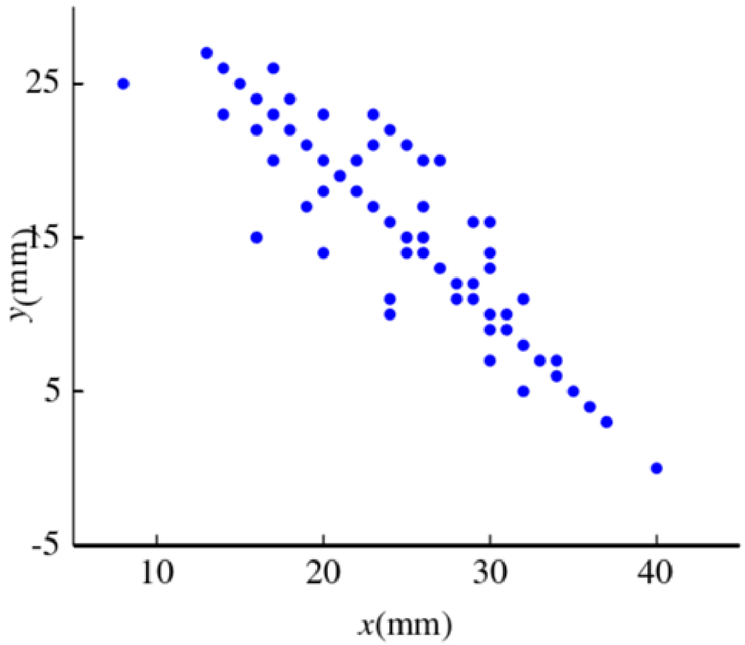

Figure S6. Workspace for symmetrical movement

● Note S5 Test bench design program

The experimental bench is shown in Figure S7a, which consists of a two-degree-of-freedom actuator platformand a transmission pivot chain (Figure S7b). The cooperation of the slide rail and carriage provides the degrees of freedom in the *y*-direction and *z*-direction during the operation of the legged robot. At the same time, its parallelism limitation ensures a low coefficient of friction in sliding with a smooth movement process. The transmission support chain utilizes a motor to provide torque, and is driven by a belt with a 1:2 transmission ratio to provide stable torque in the clockwise and counterclockwise directions periodically, and has good flexibility during the transmission process, which can ease the impact brought about by the process of walking, absorb vibrations caused by uncontrollable factors, and ensure that the lower limb structure can complete a variety of movement postures to ensure the smooth operation of the gait cycle. To assess the functional performance of the legged robot, we deployed a Qualisys three-dimensional motion capture system in the walking experiment area to obtain real-time motion data of the robot's gait (Figure S7c).

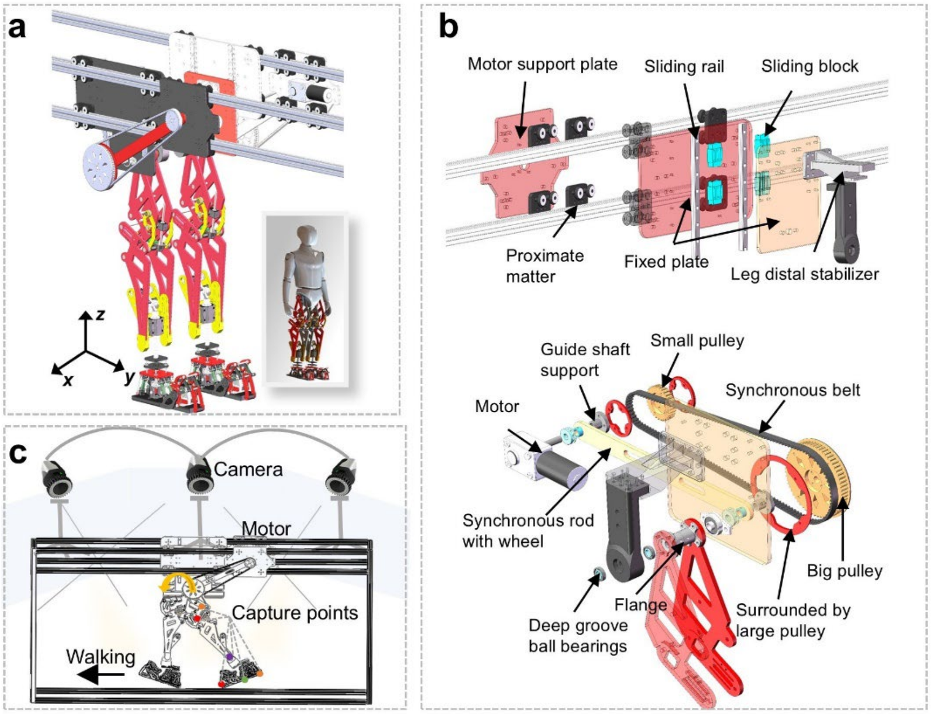

Figure S7. Stationary bipedal walking platform test conditions. a) Stationary bipedal walking platform. b) Schematic diagram of the transmission part of the walking platform. c) Conceptual diagram of legged robot locomotion and gait acquisition.

● Note S6 Control composition and circuit wiring diagram

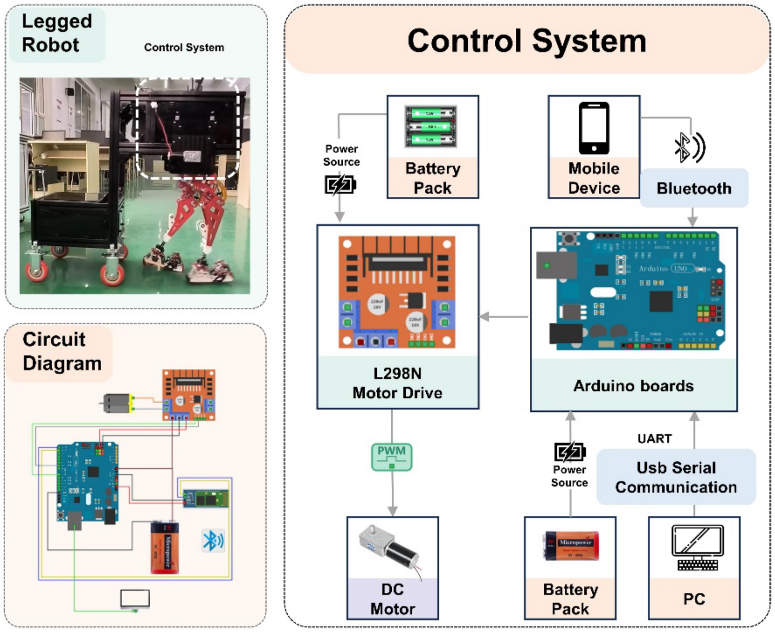

Figure S8. Control composition and circuit wiring diagram.

In this study, we employed a foundational integrated control strategy aimed at controlling the hip joint motors of the legged robot using an Arduino development board. The control algorithm was written using the Arduino Integrated Development Environment (IDE), which is capable of converting complex control logic into binary code executable on the Arduino development board. This software is responsible for generating control signals to drive the motors and achieve the predetermined gait patterns. In the system, an L298N dual H-bridge motor driver module is utilized to control the DC motors. This module can adjust the motor's speed and direction based on the input control signals. By outputting Pulse Width Modulation (PWM) signals through the digital pins of the Arduino development board, we can precisely control the motor's speed. The duty cycle of the PWM signal is used to adjust the motor's power input, enabling fine-tuned speed control. To achieve wireless control, we integrated a Bluetooth module that communicates via the RX and TX ports of the Arduino development board. This configuration allows us to remotely send speed adjustment commands from a mobile device's Bluetooth serial interface to the Arduino board. In implementing the control strategy, we adopted a driver-based programming model. By changing the duty cycle of the PWM signal, we can adjust the motor speed. This signal is generated by the Arduino's analogWrite() function and output to the motor driver module.

- **Note S7 Comparative performance validation of legged robot with tensegrity (LRT)**

The commonly used metric in the field, Cost of Transport (COT), was introduced to measure the walking efficiency of the robot. Referring to the description of COT in the literature, [1] the calculation process is as follows:

$$COT = \frac{P}{mgv} = \frac{UI}{mgv} = \frac{12V \times 1.6A}{20kg \times 9.8m/s^2 \times 0.48m/s} = 0.204 \qquad (38)$$

where:

P : average power consumption of the robot during walking;

m : mass of the robot (including the load weight of the test platform);

g : gravitational acceleration (approximately $9.8\mathrm{m/s^2}$);

v : walking speed of the robot(corresponding to $I = 1.6\mathrm{A}$).

For a comparison of the LRT with the leg structures in other studies based on the COT metric, see Table S7. For weight testing and comparison of the LRT, refer to Figure S9.

Table S7 Comparison of performance parameters of biped robots

Drive	Biped robot	Weight (kg)	Number of drive units	Cost of transport (COT)
DC motor	BRAVER[2]	8.6	6	1.01
	DURUS[3]	33.65	N/A	1.61
	Five-link robot[4]	2.975	2	0.094
	Ranger[5]	4.95	2	0.11
	NAO[6]	4.5	N/A	2.4-5.8
	LRT (This work)	0.925	1	0.204
	DRC-HUBO+[7]	7	N/A	N/A
Servo motor	KHR-2[8]	N/A	6	0.49-1.62
	DARwln-OP[9]	2.7	5	0.9-9.0
	NU-Biped-4.5[10]	7.5	7	N/A
	Airo biped robot[11]	4.7	6	N/A
	Double-jointed leg robot[12]	4.027	N/A	N/A
Cable driven	Cornell Collins biped[13]	6.35	2	0.2
	M03 biped robot[14]	1.98	6	N/A
Steering	NimbRo-	9	8	N/A

engine	OP2[15]			
	Roban robot[16]	6.6	12	N/A
Series elastic actuators	COMAN[17]	N/A	2	3.4-4.7
	SPEAR[18]	3.16	2	0.86
	Pantograph leg[19]	0.9	N/A	1.2
Hydraulic drive	Hydraulic biped robot[20]	4.64	N/A	N/A
	CVSLIP-FF biped robot[21]	7	N/A	N/A
	TaeMu biped robot[22]	6	N/A	N/A

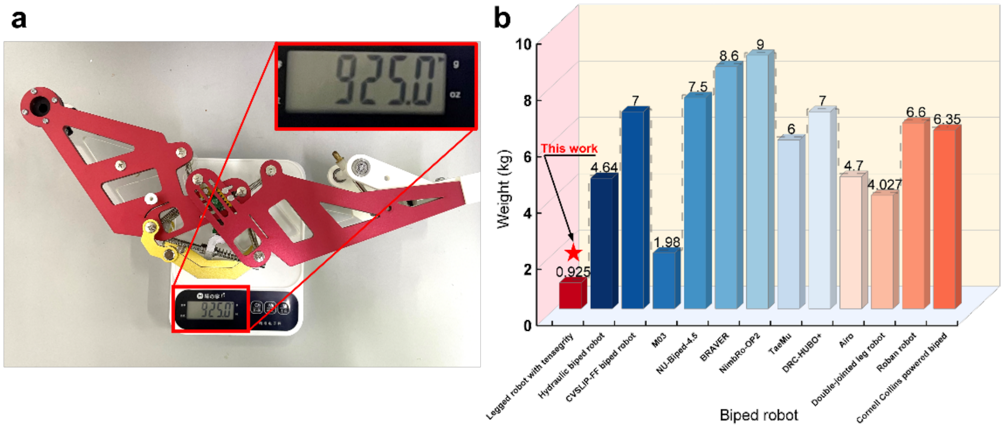

Figure S9. Comparison of leg quality of biped robot.

- **Note S8 Walking test of LRT under various road conditions**

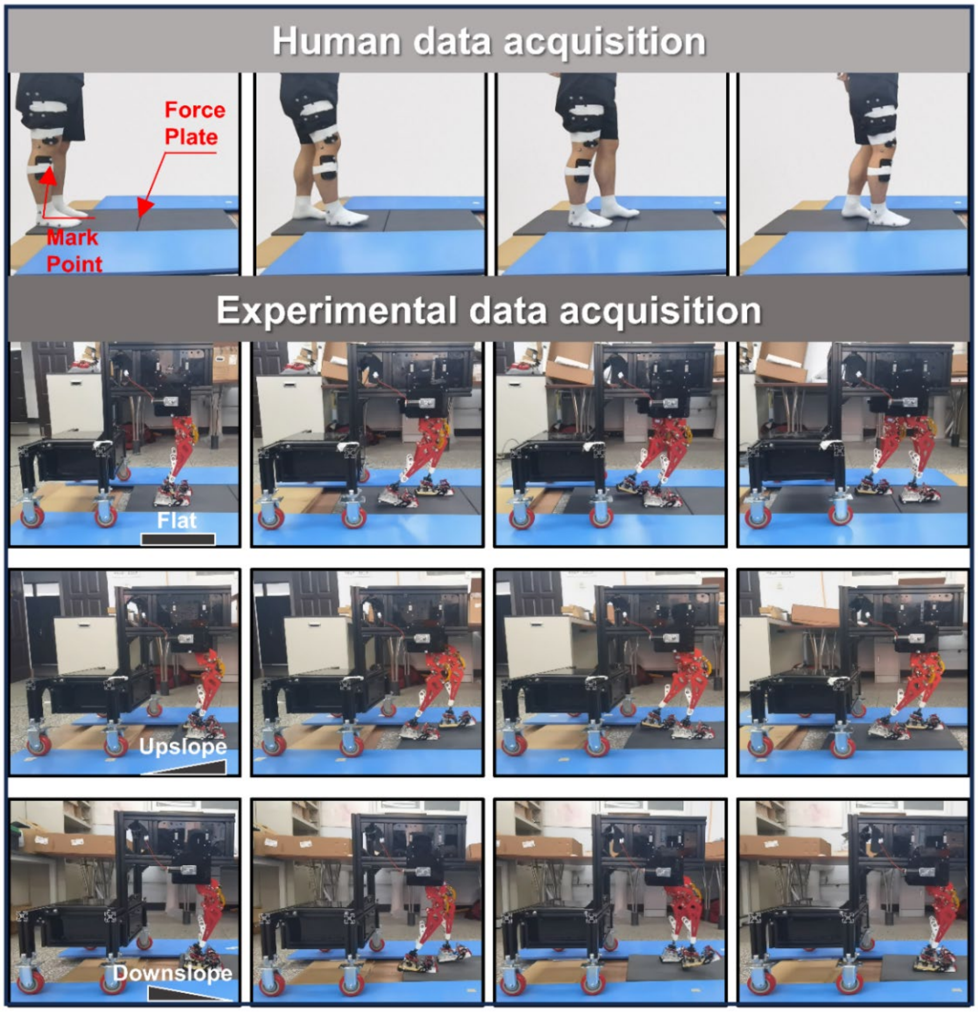

Figure S10. Experimental procedure of levelling, uphill, and downhill walking of a legged robot in comparison with human data.

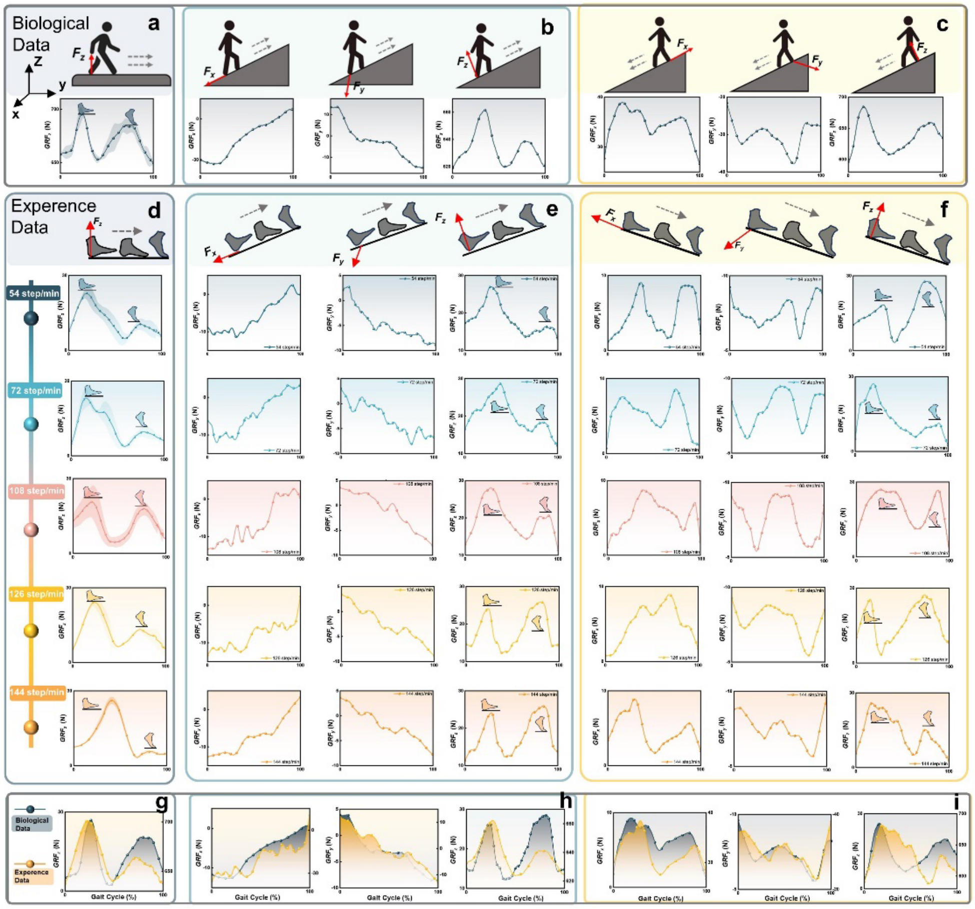

Figure S11. GRF data of the human body and LRT during level ground, uphill and downhill walking.

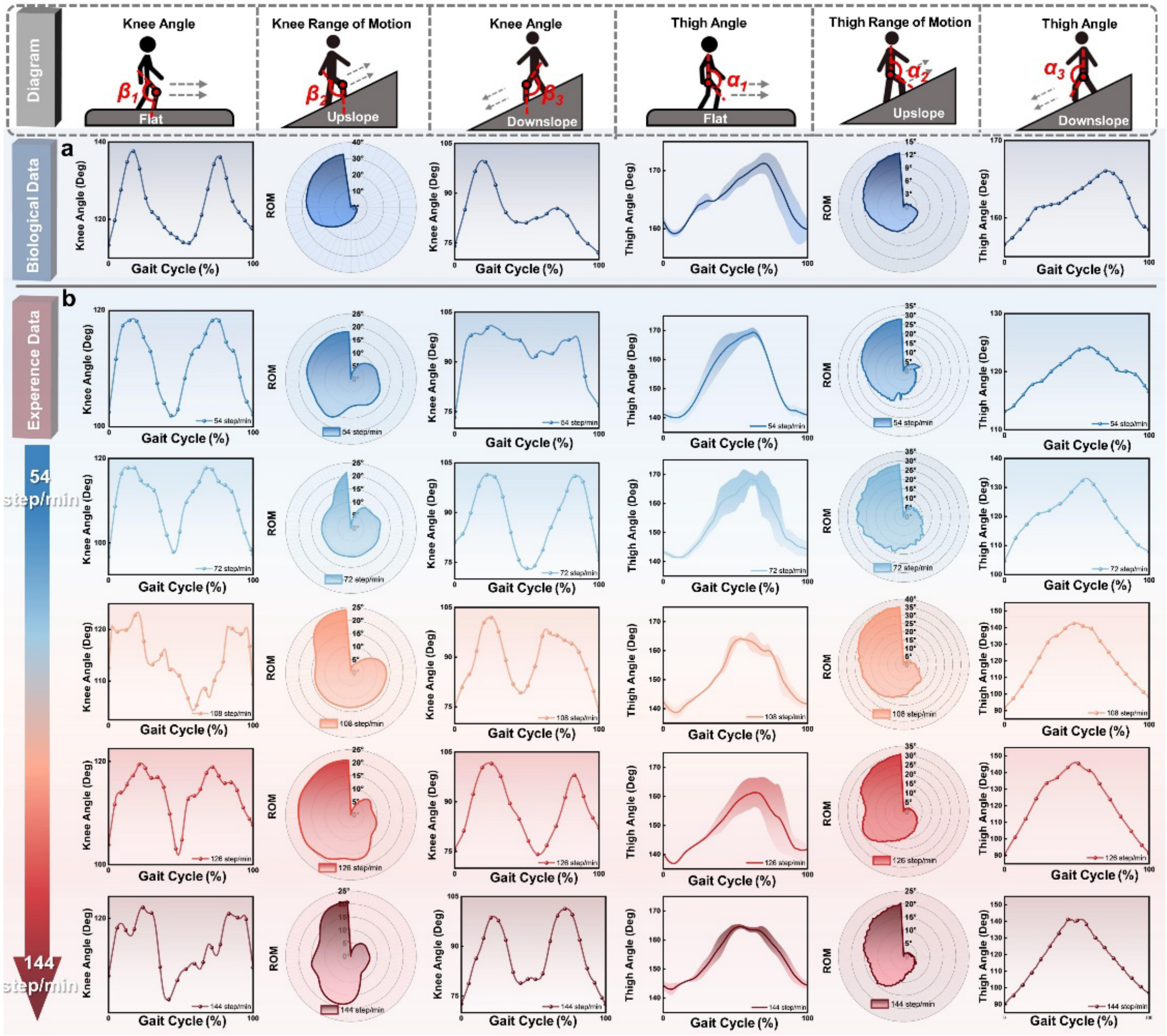

Figure S12. Joint angles and range of motion (ROM) data of the human body and LRT during level, uphill and downhill walking.

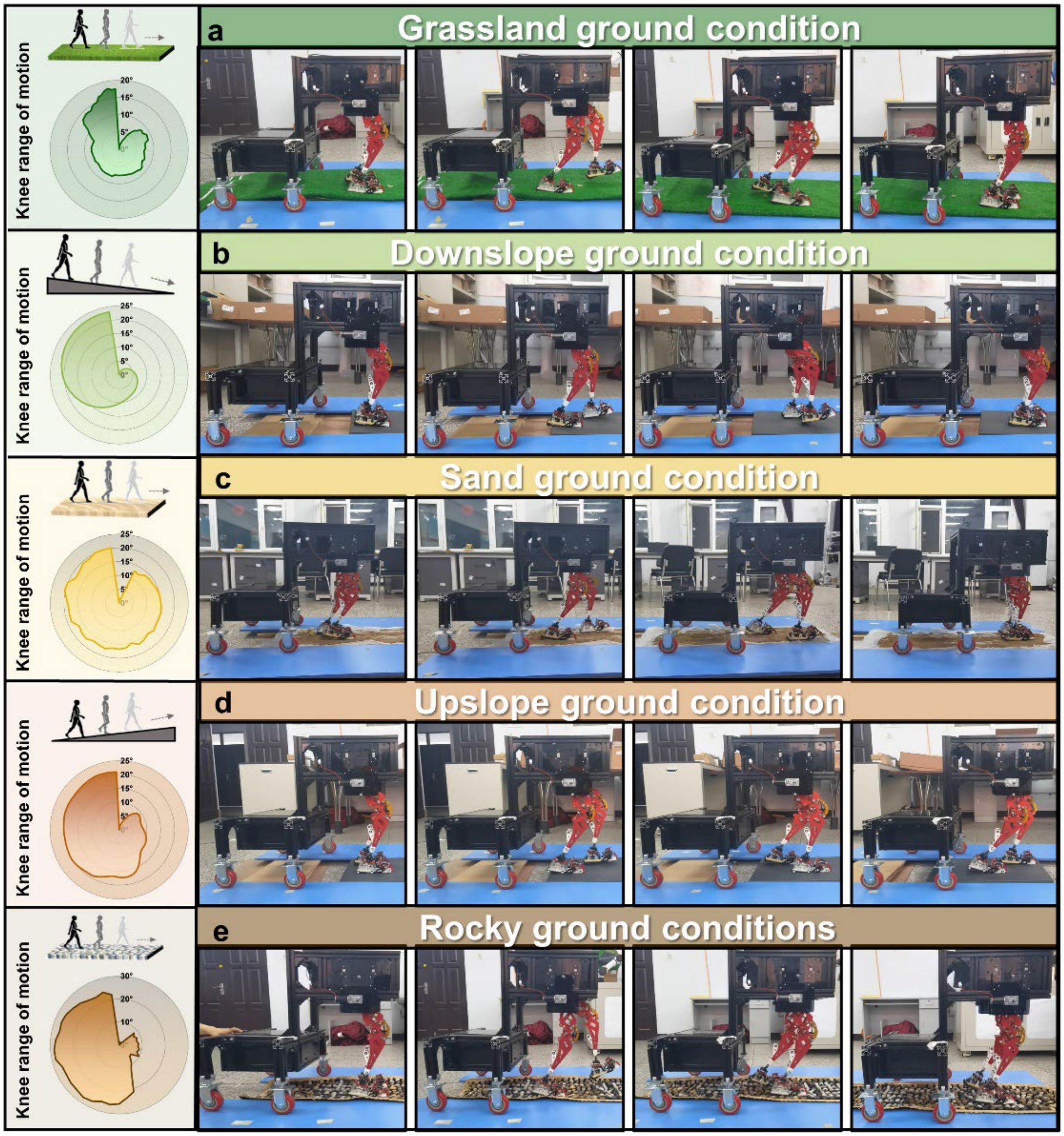

Figure S13. LRT for walking on grass, sand, rocky ground and uphill and downhill conditions.

Note S9 Motor forward and reverse phase control programme

```
// Motor Steering Control Pins
int INA1=12;
int INA2=8;
int INB1=13;
int INB2=10;
int n= 0;     // for statement judgement condition
//PWM pin
int PWM1=3;
int PWM2=9;
void setup()
{
 pinMode(INA1,OUTPUT);
 pinMode(INA2,OUTPUT);
 pinMode(INB1,OUTPUT);
 pinMode(INB2,OUTPUT);
 pinMode(PWM1,OUTPUT);
 pinMode(PWM2,OUTPUT);
 Serial.begin(9600);

}       // Initialisation
 void loop()
 {
       while(Serial.available()>0)
   {
     char c=Serial.read();
      if(c=='H')                          //First step
        {
         digitalWrite(INA2,LOW);
         digitalWrite(INB2,LOW);
         analogWrite(PWM2,0);
```

```
  digitalWrite(INA1,HIGH);
  digitalWrite(INB1,LOW);
  analogWrite(PWM1,200);
  delay(255);
  digitalWrite(INA1,LOW);
  digitalWrite(INB1,LOW);

 }
if(c=='A')                                 //First gear
 {
  digitalWrite(INA2,LOW);
  digitalWrite(INB2,LOW);
  analogWrite(PWM2,0);
  digitalWrite(INA1,LOW);
  digitalWrite(INB1,LOW);
  analogWrite(PWM1,0);

 }
  if(c=='B')                      //Second gear
  {
   for(n=0;n<=200;n++){
   digitalWrite(INA2,LOW);
   digitalWrite(INB2,LOW);
   analogWrite(PWM2,200);
   digitalWrite(INA1,HIGH);
   digitalWrite(INB1,LOW);
   analogWrite(PWM1,200);
   delay(255);
   digitalWrite(INA2,HIGH);
   digitalWrite(INB2,LOW);
   analogWrite(PWM2,200);
```

```
 digitalWrite(INA1,LOW);
 digitalWrite(INB1,HIGH);
 analogWrite(PWM1,200);
 delay(255);
 char c=Serial.read();
 if(c=='C'){ break;}
}
}
 if(c=='D')                    //third gear
{
 for(n=0;n<=200;n++){
 digitalWrite(INA2,LOW);
 digitalWrite(INB2,HIGH);
 analogWrite(PWM2,175);
 digitalWrite(INA1,HIGH);
 digitalWrite(INB1,LOW);
 analogWrite(PWM1,175);
 delay(280);
 digitalWrite(INA2,HIGH);
 digitalWrite(INB2,LOW);
 analogWrite(PWM2,175);
 digitalWrite(INA1,LOW);
 digitalWrite(INB1,HIGH);
 analogWrite(PWM1,175);
 delay(280);
 char c=Serial.read();
 if(c=='C'){ break;}
}
}
if(c=='E')                        //Four gear
{
```

```
  for(n=0;n<=200;n++){
  digitalWrite(INA2,LOW);
  digitalWrite(INB2,HIGH);
  analogWrite(PWM2,150);
  digitalWrite(INA1,HIGH);
  digitalWrite(INB1,LOW);
  analogWrite(PWM1,150);
  delay(320);
  digitalWrite(INA2,HIGH);
  digitalWrite(INB2,LOW);
  analogWrite(PWM2,150);
  digitalWrite(INA1,LOW);
  digitalWrite(INB1,HIGH);
  analogWrite(PWM1,150);
  delay(320);
  char c=Serial.read();
  if(c=='C'){ break;}
 }
}
 if(c=='F')                              //Five gear
 {
  for(n=0;n<=200;n++){
  digitalWrite(INA2,LOW);
  digitalWrite(INB2,HIGH);
  analogWrite(PWM2,100);
  digitalWrite(INA1,HIGH);
  digitalWrite(INB1,LOW);
  analogWrite(PWM1,100);
  delay(420);
  digitalWrite(INA2,HIGH);
  digitalWrite(INB2,LOW);
```

```
       analogWrite(PWM2,100);
       digitalWrite(INA1,LOW);
       digitalWrite(INB1,HIGH);
       analogWrite(PWM1,100);
       delay(420);
       char c=Serial.read();
       if(c=='C'){ break;}
      }
     }
     if(c=='G')                        //Six gear
      {
       for(n=0;n<=200;n++){
       digitalWrite(INA2,LOW);
       digitalWrite(INB2,HIGH);
       analogWrite(PWM2,75);
       digitalWrite(INA1,HIGH);
       digitalWrite(INB1,LOW);
       analogWrite(PWM1,75);
       delay(770);
       digitalWrite(INA2,HIGH);
       digitalWrite(INB2,LOW);
       analogWrite(PWM2,75);
       digitalWrite(INA1,LOW);
       digitalWrite(INB1,HIGH);
       analogWrite(PWM1,75);
       delay(770);
       char c=Serial.read();
       if(c=='C'){ break;}
      }
     }
}
```

Supplementary References:

[1] T. Mikolajczyk, E. Mikołajewska, H. F. N. Al-Shuka, T. Malinowski, A. Kłodowski, D. Y. Pimenov, T. Paczkowski, F. Hu, K. Giasin, D. Mikołajewski, M. Macko, *Sensors* **2022**, *22*, 1.

[2] Z. Zhu, W. Zhu, G. Zhang, T. Chen, Y. Li, X. Rong, R. Song, D. Qin, Q. Hua, S. Ma, *Auton. Robots* **2023**, *47*, 1229.

[3] J. Reher, E. A. Cousineau, A. Hereid, C. M. Hubicki, A. D. Ames, *Proc. - IEEE Int. Conf. Robot. Autom.* **2016**, *2016-June*, 1794.

[4] E. Selim, M. Alcl, M. Altlntas, *Robotica* **2022**, *40*, 1799.

[5] P. A. Bhounsule, J. Cortell, A. Grewal, B. Hendriksen, J. G. Daniël Karssen, C. Paul, A. Ruina, *Int. J. Rob. Res.* **2014**, *33*, 1305.

[6] D. Gouaillier, V. Hugel, P. Blazevic, C. Kilner, J. Monceaux, P. Lafourcade, B. Marnier, J. Serre, B. Maisonnier, *Proc. - IEEE Int. Conf. Robot. Autom.* **2009**, 769.

[7] T. Jung, J. Lim, H. Bae, K. K. Lee, H. M. Joe, J. H. Oh, *IEEE Trans. Robot.* **2018**, *34*, 1.

[8] J. Y. Kim, I. W. Park, J. Lee, M. S. Kim, B. K. Cho, J. H. Oh, *Proc. - IEEE Int. Conf. Robot. Autom.* **2005**, *2005*, 1431.

[9] D. Roberts, J. Quacinella, J. H. Kim, *Robotica* **2017**, *35*, 1054.

[10] M. Folgheraiter, S. Yessirkepov, T. Umurzakov, *Robotics* **2023**, *13*, 9.

[11] Y. Liu, A. Xie, S. Zhu, J. Gu, *2022 IEEE Int. Conf. Robot. Biomimetics, ROBIO 2022* **2022**, 1911.

[12] J. Babič, *2008 IEEE Int. Conf. Robot. Biomimetics, ROBIO 2008* **2009**, 155.

[13] S. H. Collins, A. Ruina, *Proc. - IEEE Int. Conf. Robot. Autom.* **2005**, *2005*, 1983.

[14] J. Tang, H. Mou, Y. Hou, Y. Zhu, J. Liu, **2024**, 1.

[15] G. Ficht, P. Allgeuer, H. Farazi, S. Behnke, *IEEE-RAS Int. Conf. Humanoid Robot.* **2017**, 669.

[16] Z. Wang, L. Kou, W. Ke, Y. Chen, Y. Bai, Q. Li, D. Lu, *Biomimetics* **2023**, *8*, 126.

[17] F. L. Moro, N. G. Tsagarakis, D. G. Caldwell, *Proc. - IEEE Int. Conf. Robot. Autom.* **2012**, 2007.

[18] X. Liu, A. Rossi, I. Poulakakis, *IEEE/ASME Trans. Mechatronics* **2018**, *23*, 2681.

[19] F. Ruppert, A. Badri-Spröwitz, *Front. Neurorobot.* **2019**, *13*, 1.

[20] G. Chen, N. Wei, H. F. Lu, L. Yan, J. Li, *J. F. Robot.* **2023**, *40*, 1525.

[21] S. Xie, X. Li, S. Lu, J. Li, C. Hu, L. Gao, *ISA Trans.* **2024**, *146*, 1.

[22] S. H. Hyon, D. Suewaka, Y. Torii, N. Oku, *IEEE/ASME Trans. Mechatronics* **2017**, *22*, 623.
